# Integrative microbial community analysis reveals full-scale enhanced biological phosphorus removal under tropical conditions

**DOI:** 10.1038/srep25719

**Published:** 2016-05-19

**Authors:** Yingyu Law, Rasmus Hansen Kirkegaard, Angel Anisa Cokro, Xianghui Liu, Krithika Arumugam, Chao Xie, Mikkel Stokholm-Bjerregaard, Daniela I. Drautz-Moses, Per Halkjær Nielsen, Stefan Wuertz, Rohan B. H. Williams

**Affiliations:** 1 Singapore Centre for Environmental Life Sciences Engineering, Nanyang Technological University, Singapore 637551, Singapore; 2 Centre for Microbial Communities, Department of Chemistry and Bioscience, Aalborg University, 9220 Aalborg, Denmark; 3 Singapore Centre for Environmental Life Sciences Engineering, National University of Singapore, Singapore 119077, Singapore; 4 Department of Civil and Environmental Engineering, University of California, Davis, CA 95616, USA; 5 School of Civil and Environmental Engineering, Nanyang Technological University, Singapore 639798.

## Abstract

Management of phosphorus discharge from human waste is essential for the control of eutrophication in surface waters. Enhanced biological phosphorus removal (EBPR) is a sustainable, efficient way of removing phosphorus from waste water without employing chemical precipitation, but is assumed unachievable in tropical temperatures due to conditions that favour glycogen accumulating organisms (GAOs) over polyphosphate accumulating organisms (PAOs). Here, we show these assumptions are unfounded by studying comparative community dynamics in a full-scale plant following systematic perturbation of operational conditions, which modified community abundance, function and physicochemical state. A statistically significant increase in the relative abundance of the PAO Accumulibacter was associated with improved EBPR activity. GAO relative abundance also increased, challenging the assumption of competition. An Accumulibacter bin-genome was identified from a whole community metagenomic survey, and comparative analysis against extant Accumulibacter genomes suggests a close relationship to Type II. Analysis of the associated metatranscriptome data revealed that genes encoding proteins involved in the tricarboxylic acid cycle and glycolysis pathways were highly expressed, consistent with metabolic modelling results. Our findings show that tropical EBPR is indeed possible, highlight the translational potential of studying competition dynamics in full-scale waste water communities and carry implications for plant design in tropical regions.

Waste water treatment plants are barriers that minimize the discharge of anthropogenic organic carbon, nitrogen (N) and phosphorus (P) to receiving water bodies that otherwise would cause eutrophication leading to severe environmental and public health problems^1^,^2^. The configuration and operational conditions of waste water treatment plants are optimized to encourage the growth of complex microbial communities capable of removing N and P^3^. Phosphorus removal is achieved by polyphosphate accumulating organisms (PAOs) that can accumulate P in excess of their growth requirement^4^,^5^. To enrich for PAOs, the biomass is cycled continuously through anaerobic and aerobic/anoxic zones. Nitrate is present as an electron acceptor instead of oxygen under anoxic conditions. The process is known as enhanced biological phosphorus removal (EBPR) and efficiently removes P from waste water without the need for chemical precipitation. Extensive research has been carried out to investigate operational conditions that favour the growth of PAOs over glycogen accumulating organisms (GAOs)^6^. GAOs are a non–PAO phenotypic group that have a metabolism similar to that of PAOs, but are unable to accumulate P in excess. Therefore, the proliferation of GAOs is thought to cause deterioration or even failure of the EBPR process. Temperature has been identified to have a significant impact on the PAO-GAO competition^6^. At temperatures >25 °C, GAOs can outcompete PAOs for organic carbon^7^,^8^,^9^,^10^,^11^. Higher GAO to PAO proportions were consistently observed in lab-scale reactors operated at 30–35 °C compared to those at 20 °C^10^,^12^. In addition, studies conducted on enriched Competibacter–GAO and Accumulibacter cultures showed evidently higher maximum acetate uptake rates of GAOs compared to those of PAOs at temperatures of 20–35 °C^7^. However, all of these findings were based on lab–scale enrichment systems, and have thus far not been verified in full–scale treatment systems.

‘*Candidatus* Accumulibacter phosphatis’ (hereafter referred to as Accumulibacter) has been reported to be largely responsible for EBPR activity observed in both laboratory reactors and full–scale plants^13^. During the anaerobic phase, PAOs hydrolyze their intracellular polyphosphate storage to provide most of the energy required to assimilate organic carbon substrates, mainly volatile fatty acids (VFAs), for the synthesis of poly–β–hydroxyalkanoates (PHAs). Additional energy and reducing equivalents are generated through glycolysis of intracellular glycogen^14^ or the anaerobic operation of the tricarboxylic acid (TCA) cycle^15^,^16^ or both^17^,^18^,^19^. In the subsequent aerobic/anoxic phase, the stored PHA is consumed to support growth, the uptake of orthophosphate and the replenishment of the internal glycogen storage. Net P removal is achieved through wastage of excess biomass containing high levels of polyphosphate.

Using the polyphosphate kinase gene (*ppk 1*) as a phylogenetic marker, Accumulibacter falls into two major types (I and II), each with multiple subdivisions (clades IA-IE, IIA-IIF)^20^,^21^,^22^. Substantial metabolic differences were detected between Accumulibacter genomes of different phylogenetic clades recovered from lab-scale enrichment reactors, suggesting potential niche differentiation between different subpopulations^23^. In addition to Accumulibacter, other organisms have been identified that exploit the alternating redox conditions in EBPR systems. Members of the actinobacterial genus *Tetrasphaera* were suggested to be putative PAOs^24^,^25^. The metabolic reconstruction from four *Tetrasphaera* genomes (*T. australiensis, T. japonica, T. elongata* and *T. jenkinsii*) showed high similarities in their polyphosphate metabolic machinery with Accumulibacter but *Tetrasphaera* has a preference for glucose as a substrate, possesses the ability to ferment substrates as an alternative source of energy and synthesizes glycogen as the storage polymer^26^. Two GAO lineages have been identified, the gammaproteobacterial ’*Candidatus* Competibacter phosphatis’ (Competibacter)^27^ and the alphaproteobacterial *Defluviicoccus*–related organisms^28^,^29^,^30^. Main differences between the assembled Competibacter-GAO and PAO genomes are found in the phosphate transport systems. The genomes of the two Competibacteraceae species sequenced to date appear not to contain genes encoding for the low affinity Pit system^31^ as in the PAOs Accumulibacter and *Tetrasphaera*, where it is required to perform P cycling^32^.

Gu *et al.*^33^ reported that the EBPR performance and stability of six temperate full-scale EBPR plants could not be correlated to high PAO abundance or a high GAO fraction. Indeed, a recent study showed sustained EBPR activity at 32 °C in a lab-scale reactor despite the dominance of Competibacter over Accumulibacter accounting for 40% and 19% of total bacteria, respectively^34^. The Accumulibacter population was dominated by a single Clade IIF. The authors concluded that Clade IIF is likely capable of outcompeting other Accumulibacter Clades and coexist with GAO. However, the well-defined operational conditions in lab-scale systems likely eliminated potential ecological niches for the growth of other Accumulibacter clades, putative PAOs and other GAOs^20^,^22^. Based on long-term operational data showing P removal activity in a full-scale water reclamation plant (WRP) in Singapore, we designed a prospective field survey, with the aim to characterize for the first time the microbial community structure and occurrence and stability of different groups of PAOs and GAOs and their associated biochemical activity in a full-scale EBPR system from a year–round tropical location. Ten sampling events were carried out over a two-month period. On the fourth sampling event, we decreased the dissolved oxygen concentration from >2.0 mg O_2_/L to <1.5 mg O_2_/L to investigate the effect of process perturbation on the stability of the EBPR community, in particular the potential interaction between PAOs and GAOs. Studies have shown that excessive aeration causes depletion of poly–β–hydroxyalkanoates (PHA) stores in PAOs^35^,^36^. Therefore, we hypothesized that increased dissolved oxygen concentration will provide a competitive advantage to GAOs over PAOs in full–scale tropical EBPR systems, a view also supported by a more recent reactor study showing that excessive aeration suppresses PAOs but will favour the growth of GAOs^37^. At each sampling event (n = 10 over 2 months), we collected samples and performed 16S rRNA gene amplicon sequencing, whole community shotgun metagenomics and metatranscriptomics, fluorescence *in situ* hybridisation (FISH), nutrient analyses and *in situ* tank measurements, and also transported biomass (activated sludge) to a laboratory setting for *ex situ* testing of EPBR activity under anaerobic/aerobic batch experiments.

## Results

### EBPR activity is detectable in tropical activated sludge

**Figure 1:**
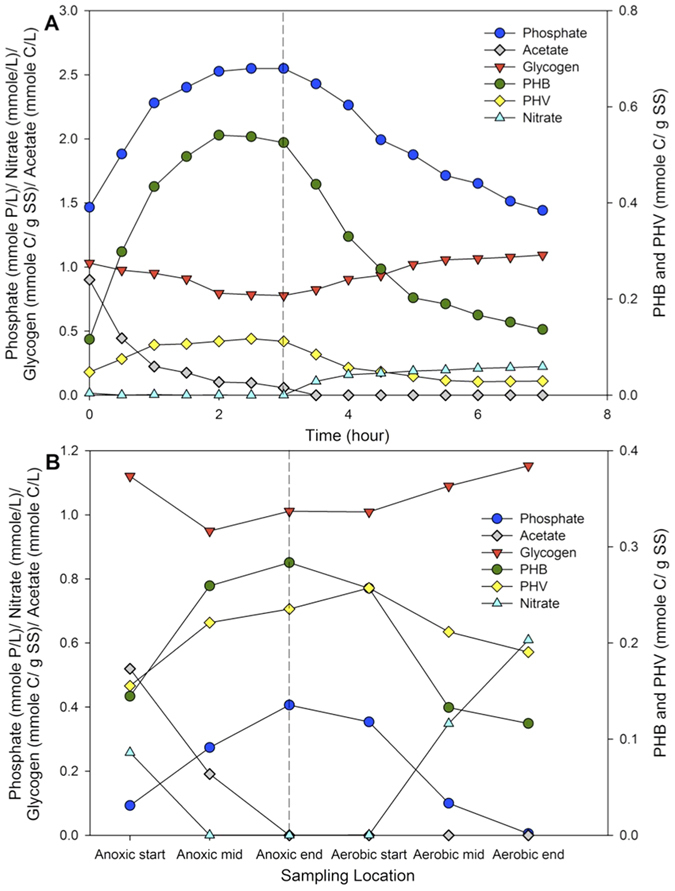
Chemical transformation occurring (**A**) in a lab-scale batch experiment conducted with activated sludge collected on the 25th July 2013 and (**B**) at Ulu Pandan South Works on the 25th July 2013. Dashed line indicates a change of redox conditions from, in (**A**), anaerobic to aerobic conditions, and in (**B**), anoxic to aerobic conditions. Anoxic condition indicates the presence of nitrate, but not oxygen, as an electron acceptor.

The presence of EBPR activity was investigated by subjecting biomass collected from Ulu Pandan Water Reclamation Plant (WRP) to cyclic anaerobic/aerobic conditions that encourage P release and uptake activity by PAOs. At all sampling time points, the collected biomass showed a typical EBPR profile in the laboratory system (Fig. 1A). Under anaerobic conditions, internal polyphosphate was hydrolyzed resulting in an increase in orthophosphate concentration in the mixed liquor. This was coupled with glycogen degradation to generate energy and reducing equivalents required for acetate uptake and PHA formation. The released P was taken up in the subsequent aerobic condition, along with PHA degradation to replenish internal glycogen storage. Similar trends were also observed in the full–scale plant where the biomass is recycled through anoxic and aerobic compartments. Despite the presence of nitrate in the anoxic compartment of the activated sludge tank, an apparent P release activity was observed, followed by P uptake in the following aerobic compartment (Fig. 1B and Supplementary Figure S1A). This was consistently observed throughout the two–month monitoring period.

### PAO and GAO taxa present in tropical activated sludge

**Figure 2:**
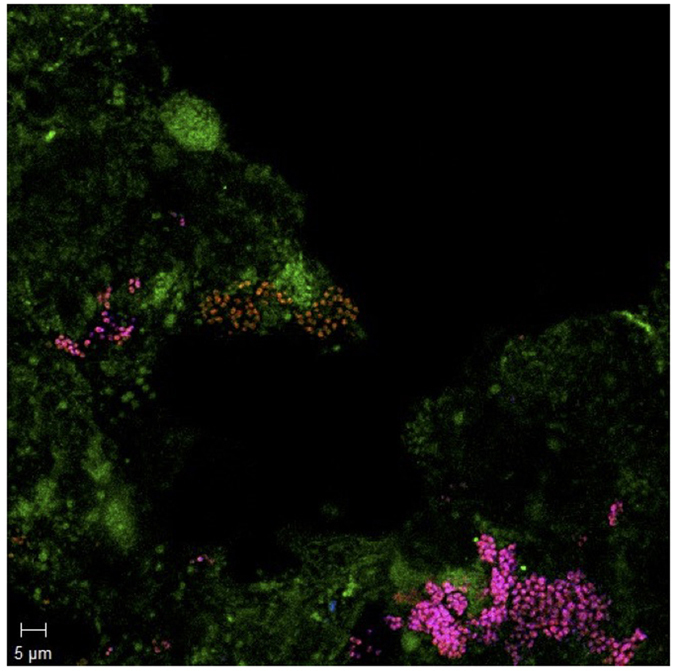
FISH image of activated sludge from Ulu Pandan South Works collected on the 25th July 2013. Bacteria hybridised with the general probe EUBMix (green), Accumulibacter–PAOs hybridized with the PAOmix (red), and Accumulibacter–PAOs Type II hybridized with ACC–II–444 (blue). Cells hybridized to both PAOmix and ACC–II–444 appear magenta.

**Figure 3:**
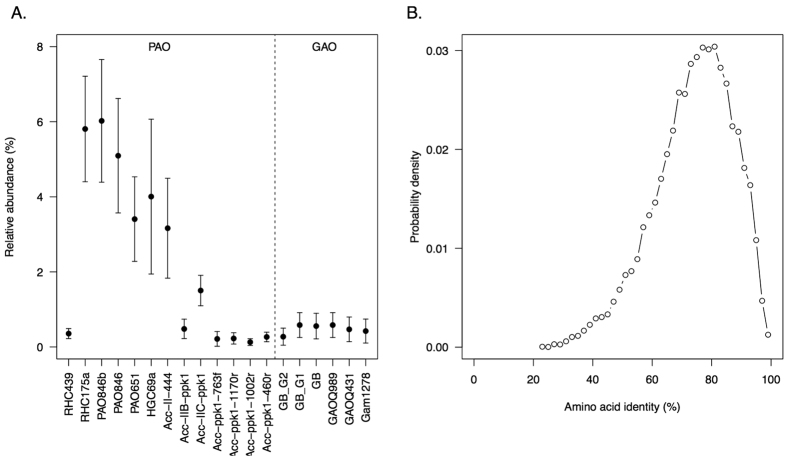
Metagenome analysis and comparative genome analysis against *Ca.* Accumulibacter phosphatis reference genome. (**A**) eFISH analysis (see Methods). gDNA read counts for instances of FISH probes from PAO and GAO (normalised to the EUB338 universal probe; see also Table S1 for relevant data). (**B**) Distribution of percent identity (amino acid identity) between ORF and proteins in the Accumulibacter reference genome.

These observations of EBPR activity would be explained by the presence of PAO species, such as Accumulibacter, the presence of which was supported by multiple lines of evidence. Epifluorescence microscopy revealed that the majority of PAO mix probe targeted cells also bound to the ACC–II–444 probe, indicating that the functional EBPR community was dominated by a close relative of Accumulibacter Type II (Fig. 2). From 16S rRNA amplicon sequencing of the community, we observed that Accumulibacter was the predominant PAO in the activated sludge, and accounted for 3.44% of total read abundance. In total there were 16 Accumulibacter–annotated OTUs detected using 16S rRNA analysis, with the three most abundant OTUs annotated to this taxon accounting for 1.74%, 0.87% and 0.65% respectively (or 3.26% collectively). We also surveyed whole community shotgun metagenome data (see Methods) to test for the presence of FISH probe and *ppk1* probe sequences from multiple PAOs, and found evidence for the presence of multiple strains, and estimated the overall PAO relative abundance to be less than 5%, consistent with the 16S rRNA amplicon sequencing results (Fig. 3A). Consistent with FISH analysis, Clades IIA, IIB and IIC were detected in all ten samples using one or more of their respective probes. Clade IID was not detected in any sample, and Clade IIF was detected in three of ten samples. Type I was detected in all samples, at lower levels than Type II probes (Fig. 3A and Supplementary Table S1).

Based on both 16S rRNA amplicon sequencing data and FISH probe sequence estimates from metagenome data, Competibacter GAOs were present at significantly lower abundances, accounting for less than 1% of all bacteria throughout the monitoring period (Fig. 3A and Supplementary Table S1). *Tetrasphaera* PAO was also present but at negligible levels with a relative abundance of 0.05%, and *Defluviicoccus* GAO was not detected in any sample.

### Modifications to EBPR activity in response to full-scale perturbation

**Figure 4:**
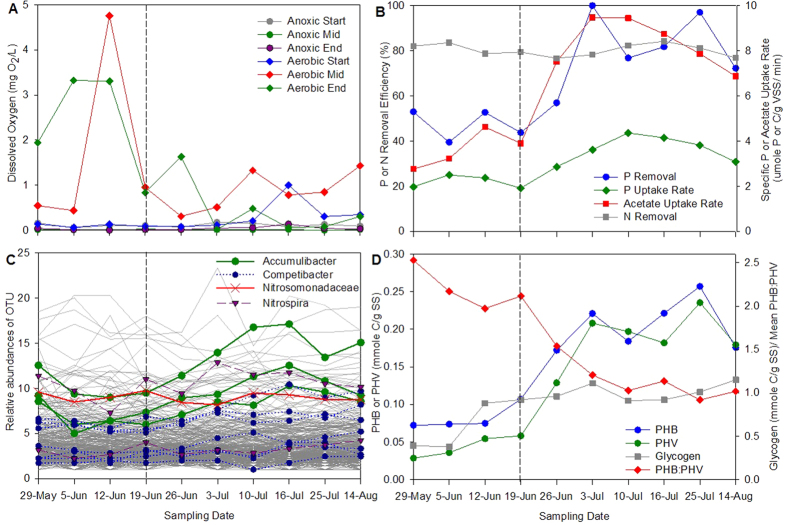
The effect of (**A**) decrease in dissolved oxygen concentration in the aerobic zone on the (**B**) phosphate and nitrogen removal efficiency at Ulu Pandan South Works and the specific phosphate and acetate uptake activity of Ulu Pandan biomass determined in lab–scale batch experiments conducted with freshly collected full–scale plant biomass; (**C**) community composition (operational taxonomic units (OTU) defined by 16S rRNA gene amplicon sequencing data); and (**D**) poly–β–hydroxyalkanoate (PHA) content and ratio and glycogen content in the biomass throughout the two month sampling period between the 29th May–14th August 2013. Relative abundances of OTUs in (**C**) are plotted on a square root scale; OTUs annotated to Accumulibacter PAO and Competibacter GAO are highlighted. Dashed line indicates the sampling point when dissolved oxygen was decreased in the aerobic zone of Ulu Pandan South Works.

The systemic reduction of oxygenation levels in the aerobic tank following the fourth sampling event permitted us to investigate the interrelationships between physicochemical state, community composition and the specific functionality of EBPR. The P–removal efficiency at the plant increased by a mean factor of 1.7 (Fig. 4B and Supplementary Figure S1) associated with the decrease in dissolved oxygen concentration from >2.0 mg O_2_/L to <1.5 mg O_2_/L (Fig. 4A). In contrast, the N-removal efficiency at the plant remained relatively stable with an average of 81% removal (Fig. 4B). The variation in EBPR activity in the full–scale setting was reproduced with freshly collected sludge in replicated lab–scale batch reactors for each sampling event (see Methods). Both the specific anaerobic acetate uptake rate and the specific aerobic P uptake rate determined from lab–scale batch experiments showed significant improvement by a mean factor of 2.3 (*P* < 0.001) and 1.7 (P = 0.002), respectively (Fig. 4B). The change point in oxygen levels also resulted in an overall 2–3 fold increase in PHB and PHV content in the sludge (Fig. 4D). In contrast to the PHA content, the glycogen content was relatively constant during the transitional period from the high to lower aeration operation (12th June–14th August) (Fig. 4D). Nevertheless, the glycogen content was observed to be substantially lower at the start of the monitoring regime during the high aeration epochs (29th May–5th June) compared to that in the following weeks (Fig. 4D). While there is insufficient data to draw concrete conclusions on the cause of this observation, we note that on the 29th of May concentrations of volatile fatty acids in the primary effluent going into the reactor were lower (Supplementary Figure S1B). Collectively, these physicochemical and activity data in both lab–scale and full–scale settings imply an increase in EBPR performance.

The substantial increase in EBPR activity was concomitant with a statistically significant increase in Accumulibacter read abundance (*q* < 0.1, see Methods), with the three main Accumulibacter–annotated OTUs increasing their relative abundance by a mean factor of 2.11 (range: 1.77–2.39). Despite having been described as competitors in EBPR systems, the relative abundance of Competibacter was always lower than that of Accumulibacter, and both taxa displayed similar dynamics in response to the decrease in oxygen levels, with the relative abundance of OTUs annotated to Competibacter increasing on average by a factor of 1.64 (range: 1.17–2.51; with 3 of the 7 consistently detected Competibacter-annotated OTUs having *q* < 0.05) (Table 1 and Fig. 4C).

In addition to these abundance changes in Accumulibacter and Competibacter, the reduction in oxygenation occurring in the fourth week was associated with a demonstrable shift in community composition, with both reductions and increases in the composition in some taxa (Fig. 4C). In total there were 2157 OTUs detectable in at least one sampling event, 337 of 2157 (15.6%) OTUs were detectable at all ten sampling events, and 43% of these demonstrated statistical support for changing their relative abundance between the high and low aeration epochs (π_0_ = 0.57; see Methods and Supplementary Figure S2), with 37 and 17 OTUs demonstrating false discovery rates of less than 10% and 5%, respectively (*q* < 0.1 and *q* < 0.05, respectively). Of the taxa in the latter category, the majority (12/17 or 71%) were only classifiable at order or a higher taxonomic rank, highlighting the fact that the majority of taxa in this tropical waste water treatment plant are largely unknown, but five OTUs for *Mycobacterium, Caldilinea, Nannocystis* and Accumulibacter were classified at genus level, and *Cytophagaceae* at family level. Changes in community abundance for altered taxa ranged between a factor of 1.33 to 3.77 (mean: 2.14) for 24 taxa that significantly increased their abundance following the reduction in aeration levels, and by a factor of between 1.51 and 12.35 (mean: 3.06) for 13 taxa that significantly decreased their abundance following reduction in aeration levels. Although the relative abundance of both OTUs detectable for *Nitrospira* increased by a factor of approximately 1.3, these changes were not statistically significant (*q*-values of 0.58 and *q* = 0.46, respectively) (Fig. 4C). Similarly, a single OTU detectable for *Nitrosomonas* did not demonstrate any significant change (*q* = 0.90) in abundance, despite the change in aeration level which corresponds with the relatively consistent N-removal performance observed in the full-scale plant.

### Characterization of biochemical activity using metabolic modelling

Examining the stoichiometric and kinetic properties of EBPR communities in the treatment plant provides a mechanistic understanding of the biochemical activities of the identified PAOs and GAOs under the investigated operational conditions. The internal carbon storage compounds in the form of glycogen and PHA are key components involved in the metabolism of PAOs and GAOs. The levels of both PHB and PHV increased following the reduction of aeration levels but the ratio of PHB to PHV decreased from 2.1 to 0.9–1.1 with the increase in EBPR activity (Fig. 4D). Acetate was the predominant volatile fatty acid detected in the primary effluent while propionate was present at lower concentrations (Supplementary Figure S1). An increase in PHV content could be associated with an increase in Competibacter abundance that converts acetate to PHV via the succinate–propionate pathway thought to be active in GAOs. However, an apparent increase in glycogen concentration was not detected as discussed above. The increase in PHV production could also be a result of increased generation of reducing equivalents by PAO either through the full or partial TCA cycle, since PHB is predicted to be the only PHA fraction produced from acetate if reducing power is generated solely through glycolysis.

The anaerobic stoichiometric data of lab-scale batch tests were compared with values obtained from metabolic models (Table 2). The anaerobic phosphorus/acetate (P/HAc) ratios were generally higher than the values predicted by the metabolic models. In addition, the anaerobic glycogen/acetate (Gly/HAc) ratios were lower than indicated in the PAO glycolysis model and the PHA/HAc ratios lie between those predicted by the glycolysis and TCA cycle model (Table 2), suggesting that Accumulibacter species within this community may utilize both TCA cycle and glycolysis to generate reducing equivalents. Using the optimized model parameters, only the Pereira model (Supplementary Figure S3), which considers the formation of reducing power by both glycolysis and full TCA cycle, could estimate the carbon fluxes of the experimental data in this study during both the high and low aeration epochs (Supplementary Table 2). It is estimated that 9% and 11% of the acetyl–CoA is oxidized through the full TCA cycle to generate on average 36% and 53% of the reducing power under anaerobic conditions during the high and low aeration periods, respectively.

### Insights from coupled metagenome and metatranscriptome analysis

From the same set of field samples studied, we conducted a coupled metagenome-metatransciptome survey to gain further insight into community composition and function associated with EBPR activity. Due to the complexity of the sampled community, we initially focused attention on studying the Accumulibacter population genome hypothesising that recovery of fragments of the corresponding genomes would be possible from a metagenome assembly, and these could be used to examine the degree of divergence from the Clade IIA str. UW-1 complete reference genome^38^. From a metagenome co–assembly constructed from whole community gDNA from all 10 samples (see Methods, Supplementary Results and Supplementary Table S3), we identified 42,349 genes (open reading frames) that held consensus (lowest common ancestor) annotations to Accumulibacter at genus level with a DIAMOND bitscore threshold of >50 (see Methods). These contigs resided on 6888 contigs, of which 2203 had all cognate ORFs annotated to genus *Ca.* Accumulibacter under our inclusion criteria. Compared against the Clade IIA reference genome, we can observe at least one instance of 3396 of 4562 coding genes in that reference genome (gene recovery rate, 74.4%) at this level of stringency. The mean level of amino acid identity (AAI) to Clade IIA reference coding sequences was 78.5% (median: 80%), with 3.5% having >95% identity (Fig. 3B), although recoverable genes associated with plasmids had a higher mean identity (87.3–90.1% mean-AAI in plasmids, as distinct from 78.1% on the chromosome). When we expanded the AAI-analysis to include a further 8 draft Accumulibacter genomes available from NCBI, we observed that the distributions of AAI in this set of ORFs showed a higher degree of identity with Type II annotated genomes, particularly Clade IIC, and less with Type I sequences (Fig. S4 and Supplementary Table S4). Therefore these results suggest that the most abundant taxa are closely related to a Type II genome.

Recognising the limitations of homology search to precisely pinpoint taxonomic identity, we performed an independent analysis of contigs from our co-assembly using the metagenome binning software MetaBAT^39^, and then employed CheckM^40^ to make taxonomic classifications of those bins. Restricting attention to the 387,184 contigs of length greater than 1500 n.t., this analysis defines a total of 436 bins, of which seven were classified to taxonomic identifier UID3971 which is selective for *Ca.* Accumulibacter and some *Rhodocyclaceae*^40^. Five of the seven UID3971-annotated bins harboured high proportions of ORFs annotated to *Ca.* Accumulibacter as defined above (see Supplementary Data File 1). We observed that one bin (*binsp1500.28*; Supplementary Data File 1) was enriched for Accumulibacter annotated genes but was not annotated to UID 3971 (*i.e.* it contained no marker genes as defined by CheckM), and two UID3971-annotated bins showed a greater level of association with ORFs annotated to *Dechloromonas aromatica* than to Accumulibacter (*binsp1500.28* and *binsp1500.60*; Supplementary Data File 1).

We also applied the same analysis to ORFs classifiable to genus *Ca.* Competibacter, but only identified 1198 ORFs that were contained on only 197 contigs, of which only 20 had all cognate ORFs annotated to genus *Ca.* Competibacter. We could not identify any MetaBAT-derived bins associated with Competibacter using either CheckM-results or from our ORF-level analysis.

We also examined whether changes in contig abundance between epochs of high and low aeration were consistent with 16S data, using data from an independent set of samples co-collected with samples used to generate the co-assembly, in order to avoid baises or artefacts related to data re-use (see Methods). From the cohort of Accumulibacter–annotated ORFs, and also in Accumulibacter associated bins, we observed an average increase in abundance of a factor of 1.41 (range 1.15 to 1.77) following the transition to low–aeration conditions, consistent with the change detectable in these taxa with 16S amplicon sequencing, albeit at a reduced magnitude of change in abundance (Methods, Supplementary Results and Supplementary Figure 5).

We then analysed the associated metatranscriptome data from these samples to gain further insight into functional (mRNA level) changes associated with Accumulibacter taxa, examining both the overall level of expression across samples, as well as any changes associated with the transition from high to low–aeration epochs. We confirmed that all EBPR genes previously studied by He *et al.*^41^ were expressed, and noted they were collectively expressed at significantly higher levels than other genes in the Accumulibacter population genome in this community (Mann Whitney *U*-test, W = 74574, *P* = 1.5 × 10^−7^). We then focused on analysing data at a pathway level in order to gain a degree of mechanistic insight. We first ranked canonical KEGG pathways, annotated to Accumulibacter genes, by their overall level of expression across all samples (see Methods). The pathway with the highest expression level was *synthesis and degradation of ketone bodies* (KEGG: app00072), which contains primary genes in the canonical PHB processing pathway, namely β-ketothiolase (CAP2UW1_2144) and acetyl-CoA acetyltransferase (CAP2UW1_3189), and acetoacetyl-CoA reductase (CAP2UW1_3919). Additionally, a gene containing poly-beta-hydroxybutyrate polymerase domains (CAP2UW1_3191), whose role is consistent with converting (R)-3-OH-Butyryl-CoA to PHB, was also detected, albeit at lower levels of expression. Further pathways related to VFA metabolism were also among the highest expressed in the Accumulibacter pan-genome, namely *propanoate metabolism* (KEGG: app00640), *butanoate metabolism* (KEGG: app00650) and *pyruvate metabolism* (KEGG: app00620) were also highly expressed relative to other metabolic pathways. We also used these data to gain further insight into the nature of reducing power, specifically by comparing the overall expression level of the *TCA Cycle* (KEGG: app00020) and *Glycolysis/Gluconeogenesis* (KEGG: app00010). Genes that encode proteins that are members of either the TCA and/or glycolysis pathways were expressed at high levels relative to most other genes (Supplementary Table S5); with each of these pathways being ranked 7th and 10th in terms of overall expression level compared to 104 KEGG pathways defined in this analysis. Despite the difference in ranking using this expression statistic, there was no consistent statistically significant difference in the distribution of expression levels between the two pathways (see Supplementary Results). Therefore, at the level of gene expression, both pathways appeared to be active, supporting the notion that the PAO sub–community studied here utilizes both pathways during EBPR.

We also examined these data (Supplementary Results) to determine whether functional changes between high and low aeration epochs were detectable, and whether any changes in pathway expression between the two epochs were consistent with the biochemical changes reported above. We first performed an analysis of gene expression changes in genes associated with Accumulibacter–annotated OTUs, and demonstrated that a positive increase in the gene expression was observable above the expected statistical background (Supplementary Figure S6A): an observation that is consistent with the increase in the abundance of Accumulibacter detected above, and highlights that the systemic change in aeration conditions influences functional activity at the transcript level. Once these changes in abundance were taken into account, we examined which pathways showed evidence of differential expression between high- and low-aeration epochs: interestingly, the results of this analysis suggest that most of the key pathways discussed above did not change their relative expression (Supplementary Figure S6B), consistent with the concept that the changes in EBPR function we observe appear primarily related to a change in taxon abundance, with corresponding maintenance of overall physiological state.

## Discussion

In this study we have characterised for the first time the community composition and dynamics of a full-scale tropical activated sludge plant performing EBPR. Tropical EBPR has long been considered unfeasible because GAO–organisms will outcompete PAO–organisms for organic carbon at the higher temperatures encountered in tropical conditions^6^. Using a systematic investigation of a waste water treatment plant in Singapore and employing multiple lines of evidence, we show that this explanation is not necessarily true, and that a functioning PAO community can co–exist with GAO species, with a higher observed abundance of the former compared to the latter. We demonstrate that EBPR exists in a tropical waste water treatment system using standard physicochemical surveys. Specifically, the anaerobic acetate uptake rates of the tropical sludge studied here are comparable to those of two EBPR plants with an average operating temperature of 22 °C, with acetate uptake rates ranging from 0.0042 to 0.0210 mmol C/g VSS/min^42^. The aerobic P uptake rates of 0.0015–0.0017 mmol P/g VSS/min reported by Pijuan *et al.*^42^ are actually lower than those reported here. Our observations are supported by field and laboratory protocols, each performed on the same volume of sampled biomass using a time series design extending over two months, and which clearly demonstrates that EBPR activity is consistently present in this microbial community.

An important component of our study involves a controlled manipulation of aeration levels in the full-scale plant, made by dropping the oxygen levels from from >2.0 mg O_2_/L during the first four sampling weeks to <1.5 mg O_2_/L in the subsequent six sampling weeks. We undertook this perturbation based on previous evidence^35^,^36^ suggesting that lower aeration may help PAOs to compete with GAOs, and find compelling support that the genus Acccumulibacter, among other taxa, did indeed increase in abundance in the lower aeration epoch, Despite being limited to ten sampling events, we show using a rigorous statistical analysis, supported by simulations and supplementary analyses (confirming the absence of artifacts commonly found in time series analyses that would increase the false positive rate), that we can detect these effects with a high degree of confidence.

In our analysis, we identified a close relative of Accumulibacter whose abundance and activity is correlated with demonstrable changes in EBPR activity. Predicted Accumulibacter genes show an average amino acid identity to the Clade IIA reference sequence^38^ of around 80%, suggesting that the taxonomic relationship probably exists at the intragenus level^43^,^44^, and comparison with more recent draft genomes^23^ further supports a close relationship to Type II strains, particularly Clade IIC. The complexity of the metagenome of this unmodified community does not permit the recovery of the full genome sequence of these species, consistent with the known limitations of current metagenome analyses^45^; however, using estimates of relative taxon abundance from 16S rRNA amplicon sequencing in combination with a recently published model of metagenome coverage^46^, we can conclude that we have sequenced to a depth that permits in–principal recovery of full genome sequences from these taxa. Therefore, gene content differences compared to the reference genome are likely to be attributable to either genuine differences in genome composition or undetermined, poorly understood experimental factors that influence gene recovery, and less likely to result from low abundance. Proteomics data from a lab-scale EBPR system showed that the co-occurrence of Accumulibacter strain variants displaying different phenotypes likely enhances process performance^47^. Similar findings have also been reported in other ecosystems indicating the importance of micro and macro diversity in the overall ecosystem functioning^48^,^49^. In this study, both 16S rRNA gene amplicon and eFISH analysis suggest that multiple species or sub–species are present, and the recovery of the genome(s) of these taxa, and dissection of the related structural and functional microheterogeneity using enrichment systems and single cell genomics, is now a priority. Although the known PAO *Tetrasphaera* has also been found to be highly diverse and abundant in full–scale EBPR plants in temperate climates^50^,^51^, this genus was present at relatively low abundance at Ulu Pandan WRP supporting the postulation that Accumulibacter and *Tetrasphaera* each predominate in different niches^26^.

A key finding of this study concerns the apparent co–existence of PAO and GAO species, rather than their expected competition as predicted from previous studies. In the present community, Competibacter was the only known GAO detected, whereas *Defluviicoccus* was not detectable. Interestingly, while Competibacter is detectable in our community, our analysis suggests that the genomic backbone of these organisms is likely to be considerably different from that of other members of this genus whose draft genomes are currently available. In addition to temperature, numerous environmental conditions such as pH, COD:P ratio, type of carbon source and dissolved oxygen concentration and aerobic hydraulic retention time have been shown to affect the competitive relationship between PAOs and GAOs^6^,^37^. Using enriched cultures of Accumulibacter PAOs and Competibacter GAOs, Carvalheira *et al.*^37^ demonstrated that GAO activity decreased at low dissolved oxygen concentration whereas PAOs maintained their aerobic activity resulting in an overall increase in the PAO fraction^37^. In addition, GAOs thrived compared to PAOs when the aeration length was increased and dissolved oxygen concentration maintained at 2 mg O_2_/L, decreasing EBPR activity^37^. In our study, we have demonstrated that lab–derived hypotheses in regard to temperature and dissolved oxygen selection of GAOs were falsified when examined *in situ* in a tropical climate. Over the two–month monitoring period, Accumulibacter PAOs were at all times present in higher proportions than Competibacter GAOs, and furthermore these taxa displayed an analogous response to the decrease in dissolved oxygen level, suggesting that they were not competing with one another. This is in contrast to lab–scale reactor systems, in which PAOs and GAOs are co–enriched due to the similarities in their metabolic capacity to thrive under cyclic redox conditions.

Similar to Accumulibacter, 16S rRNA amplicon analysis also showed the presence of multiple Competibacter strains. Environmental gradients contribute to fine–scale diversity and niche differentiation^52^. In contrast to highly controlled conditions in lab-scale systems, the wide range of substrate available in waste water and diurnal variation in hydraulic and nutrient load in full-scale systems may play a role in providing greater niche partitioning that supports the co-existence of multiple PAO and GAO strains. In our unmodified field community studies, up to 43% of member taxa changed abundance in response to the decrease in oxygenation level. It is possible, therefore, that some of these changes resulted from previously unrecognized interactions between PAO and/or GAO species, relationships that have so far been ignored due to limitations of current enrichment methods. This highlights the need for experimental platforms that can permit the mechanistic investigation of potential species interactions identified in surveys of unmodified field communities.

One important metabolic difference between PAOs and GAOs is the source of energy and reducing power necessary for VFA uptake and PHA synthesis under anaerobic conditions. Glycolysis is the primary source of energy and reducing power for GAOs, whereas PAOs utilize poly-P to provide energy and the TCA cycle or glycolysis or both to generate reducing power^53^. Based on our anaerobic stoichiometric data and results of metabolic modeling analysis, we predict that glycolysis and TCA cycle are of equal importance in supplying reducing power to Accumulibacter in this activated sludge community under anaerobic conditions. This prediction is also consistent with metatranscriptome data, demonstrating that genes in both pathways were highly expressed and at approximately the same overall levels (see Supplementary Results for further dissection of this point). These observations are entirely consistent with previous genomic data in the Clade IIA reference showing the presence of genes encoding for glycolysis and the operation of the full and split TCA cycle^38^, and proteomic data from mixed Accumulibacter enrichment (containing Type I and Clades IIA, IIB, IIC and IID), which detected proteins associated with the TCA cycle being able to anaerobically generate reducing equivalents^18^. The contribution of the TCA cycle estimated in this study is higher than that found in an Accumulibacter enrichment that estimated TCA contributing approximately 25% of the necessary NADH under non–glycogen limited conditions^19^. In addition, the contribution of the TCA cycle increased under the low aeration condition, likely because more of the PHA was used for biomass generation than for glycogen production^54^. This is supported by an overall increase in Accumulibacter abundance, whereas glycogen content in the biomass remained relatively constant during the transition period from high to low oxygenation level. The higher P/HAc ratio compared to that of the metabolic models also indicates that the TCA cycle was active^54^ and that lower glycogen degradation resulted in higher levels of P release being required to compensate energy requirement for acetate uptake^19^,^55^,^56^. The dependency on polyphosphate and TCA cycle suggests that the glycogen levels in Accumulibacter biomass were limiting and insufficient to fully support the ATP and reducing equivalents required for organic carbon uptake and PHA production by PAOs.

Glycogen can be limiting especially under full-scale settings due to the low availability of external organic carbon substrate or longer cycle time compared to that in lab-scale reactors leading to depletion of glycogen storage^55^. Gu *et al.*^33^ reported that a high readily biodegradable chemical oxygen demand (rbCOD) to P ratio promotes the proliferation of GAOs. Out of the six investigated full-scale EBPR plants, *Competibacter* and tetrad forming organisms such as *Defluviicoccus* were found in all plants except for one receiving wastewater with the lowest rbCOD to P ratio, suggesting that excess carbon is crucial to support GAO type metabolism. In addition, the ability to utilize the TCA cycle to generate reducing equivalents may also explain the dominance of Accumulibacter Type II over Type I that relies mainly on glycolysis. However, the experimental aerobic P/PHA yields were significantly higher than the PAO model predictions, indicating that total phosphorus uptake could include contributions from PAOs other than Accumulibacter^55^. Tu and Schuler^57^ concluded that the importance of GAOs as a cause of EBPR failure may have been overestimated. A low reactor organic substrate concentration resulted in the dominance of Accumulibacter despite low pH conditions of 6.4–7.0, previously thought to favor the growth of GAOs^57^. The volatile fatty acids concentration in the anoxic compartment of the plant was consistently below 2 mg/L. Even though the maximum acetate uptake rate of GAOs is higher than that of PAOs at warmer temperatures (25—35 °C)^7^, PAOs may be able to scavenge low acetate concentrations efficiently using active transport mechanisms^58^.

Current understanding of the EBPR process under tropical (high temperature) conditions is largely based on lab-scale enrichment studies using sludge originating from temperate-region treatment plants as inoculum. Here we conclusively show it is possible for EBPR to function in tropical climates, that the key organisms known to be responsible for EBPR are present, and that they are able to flourish in an activated sludge community in a full-scale tropical treatment plant. We show these organisms are closely related at a genome level to relevant species from temperate regions with comparable biochemical activity, and that their abundance and activity are highly correlated with EBPR activity. In ongoing work, we are investigating the mechanistic basis for this phenomenon, with the aim of providing specific recommendations that could inform routine implementation in full-scale operational conditions. Collectively, these observations provide unambiguous scientific support for the position that EBPR can be routinely implemented in wastewater treatment plants in tropical regions, in order to mitigate the immediate impact of excessive P in surface waters, as well as to facilitate enhanced sustainable development, for example, in regard to future P-recovery.

## Methods

### Field sampling and lab–scale batch experiments

Ulu Pandan Water Reclamation Plant (South), located in south–western Singapore receives domestic waste water at a rate of 200,000 m^3^ per day. The plant consists of primary clarifiers and six lines of activated sludge treatment. After primary sedimentation, waste water is separately introduced into parallel activated sludge units, each consisting of a typical modified Ludzack–Ettinger (MLE) system for biological nitrogen and carbon removal. The working volume of each MLE system is 5680 m^3^ (Supplementary Table S7).

Ten sampling events were carried out over a two–month period from the 29th of May to the 14th of August, 2013. In the first four sampling weeks, the dissolved oxygen concentration at the end of the aerobic zone was maintained above 2 mg O_2_/L and adjusted to less than 1.5 mg O_2_/L in the subsequent sampling weeks. Each sampling event was conducted at the same time of the day (10:00 am) to ensure that the monitored conditions were under a similar influent loading rate. On each sampling event, liquid samples were collected from the primary effluent and six locations in the MLE tank, namely start, mid and end of the anoxic zone and start, mid and end of the aerobic zone (Supplementary Figure S7). The pH, temperature, redox potential and dissolved oxygen concentration at the six within–tank sampling locations were also measured with a portable dissolved oxygen/pH/EH/T meter (YSI Professional Plus, United States), at the same time of liquid phase sampling. The collected liquid samples were filtered with 0.22-μm disposable sterile filters and analyzed for ammonium, nitrate, nitrite and orthophosphate using ion chromatography (Prominence, Shimadzu). Filtered primary effluent samples were also analyzed for volatile fatty acids using gas chromatography (Prominence, Shimadzu). Non–filtered liquid samples collected from the primary effluent were immediately acidified with sulfuric acid and analyzed for total phosphorus (TP) and total chemical oxygen demand (TCOD). Mixed liquor samples were collected from the mid aerobic zone for mixed liquor suspended solids (MLSS) and volatile solids analyses (MLVSS). TCOD, TP, MLSS and MLVSS analyses were carried out based on Standard Methods^59^. Mixed liquor samples were also collected from the six within–tank sampling locations, fixed for at least 1 h (8% formaldehyde), washed in 0.9% NaCl, then freeze dried before poly–β–hydroxyalkanoates (PHA) and glycogen analyses. PHA analysis was performed as described in Oehman *et al.*^60^ to detect polyhydroxybutyrate (PHB) and polyhydroxyvalerate (PHV) and glycogen analysis as described in Kristiansen *et al.*^26^. Samples for DNA extraction and fluorescence *in situ* hybridization (FISH) were collected from the mid aerobic zone. Samples for DNA extraction were snap–frozen in liquid nitrogen and stored at −80 °C. Samples for FISH were immediately fixed in 4% paraformaldehyde.

For each sampling day, batch tests were conducted with freshly collected recycled activated sludge. Two litres of sludge were diluted with 2 L of synthetic medium for each batch test to a final concentration of approximately 1.5–1.8 g/L volatile suspended solids (VSS). The sludge was subjected to a sequence of anaerobic (3 h)/aerobic (3 h) conditions in duplicate lab–scale sequencing batch reactors. In all batch tests, the pH was controlled at 7.0 ± 0.2 with 0.5 M of NaOH and HCl and the temperature maintained at 30 °C. At the start of the anaerobic phase of each batch test, the sludge was sparged with nitrogen for an hour to remove all dissolved oxygen. Oxygen was maintained close to saturation during aerobic phases. The mineral medium used was similar to that in Nittami *et al.*^61^ with an acetate concentration of 10–15 mg C/L and a phosphate concentration of 40–45 mg P/L for a C:P molar ratio of 1:1. The concentration of the nutrients was optimised so that it would be possible to achieve in a 4–6 h period the maximal utilisation of the PHA internal reserves of PAOs (or GAOs) as recommended in Oehmen *et al.*^62^, obtained when a stabilisation of phosphorus levels was observed. Samples were periodically taken for analysis of acetate and orthophosphate concentration in the supernatant and to determine PHA and glycogen in the biomass. Mixed liquor (ML) samples were also taken at the beginning and end of each phase for the determination of the MLSS and MLVSS concentrations.

### Nucleic acid extraction, library preparation and sequencing

Full protocols for these procedures are provided in Supplementary Methods. Briefly, DNA was extracted using the FastDNA™ 2 mL SPIN Kit for Soil (MP Biomedicals, USA), optimised for DNA extraction from activated sludge^63^ (see Supplementary Methods 1.1). From this extraction, 16S rRNA gene amplicons were generated for a ~500 bp fragment from the variable V1 to V3 region and sequenced on an Illumina MiSeq (see Supplementary Methods 1.3). For gDNA, sequencing library preparation was performed using a modified version of the Illumina TruSeq DNA Sample Preparation protocol, with sequencing performed on an Illumina HiSeq2500 sequencing run at a read–length of 151 bp paired–end (see Supplementary Methods 1.4). RNA extractions were performed with Zymo Research ZR Soil/ Fecal RNA Isolation Kit (Catalog #R2040) with some protocol modifications (see Supplementary Methods 1.2). RNA purity was determined by Thermo Scientific Nanodrop 2000 by calculating an A260/A280 and A260/A230 to screen for contaminants present in purified RNA. The concentration of extracted RNA was determined by a qubit 2.0 fluorometer, and RNA integrity (RIN) was measured using the Agilent 2200 Tapestation system, prior to library preparation and sequencing on an Illumina 2500 using a 101 bp paired–end run (see Supplementary Methods 1.5). All raw metagenome, metatransriptome and amplicon sequencing data used in this study are publicly available from NCBI under BioProject ID: PRJNA320780 (http://www.ncbi.nlm.nih.gov/bioproject/320780).

### Analysis of sequencing data

Full details of all data processing and analysis procedures for 16S rRNA amplicon, metagenome and metatransciptome data are provided as Supplementary Methods (Sections 1.6–1.8). Briefly, for 16S rRNA data, read 1 was trimmed to 200 bp of high quality and the sequences were clustered into OTUs (97%) and classified using QIIME (v. 1.8)^64^ with GreenGenes (13/5) as the reference database^65^. Competibacter was manually curated based on the MiDAS taxonomy (midasfieldguide.org)^66^. Samples were randomly subsampled to an even depth and compared using the R^67^ package Phyloseq^68^. We constructed read count matrices using these uniformly sampled read data and further normalised these using log base 10 transformation of (unity offset) pseudo–counts. The relative abundance of each OTU was estimated by summing the total number of associated reads and expressing this number as a quotient to the total number of included reads. Relative abundances were summed across multiple OTUs annotated to the same taxa. To test differences between the two epochs with high and low levels of aeration, we used a test statistic designed to measure step–like changes in the mean level of time series data from each OTU^69^, with correction for multiple comparisons being made using the Storey–Tibshirani estimator^70^ (Supplementary Figure S2), after confirming using simulation that we had adequate power using only 10 samples split into groups of 4 and 6 (see Supplementary Figure S8), and that the false positive rate was not artifactually increased by autocorrelation^71^,^72^ (Supplementary Figure S9). For metagenome data, raw reads were quality trimmed and adapter removed using cutadapt–1.2.1^73^. We generated a co–assembly from gDNA data from each of the ten sampling events using SOAPdenovo (SOAPdenovo–63mer from SOAPdenovo2–linux–version r223) with default parameters^74^. Open reading frames (ORFs) were predicted using MetaGeneMark^75^, and annotated to RefSeq–NR (September 2015) using DIAMOND^76^. Taxonomic assignments to ORFs were processed using a command line implementation of the MEGAN lowest common ancestor algorithm (*lcamapper*, D.H.H and X.C)^77^, using default parameters (min score = 50, top percent = 10 and max expected = 0.01). We extracted ORFs that were assigned to species *Ca.* Accumulibacter, and extracted the corresponding DIAMOND hit results selected by *lcamapper*, and from these, calculated the average percent amino acid identity against the reference sequences for this taxon. For binning analysis, we used. BAM files generated by SOAPdenovo as input to MetaBAT^39^, and contigs assigned to each bin were subsequently analysed using CheckM^40^. For contig and bin level analysis using ORF taxonomic assignments (as calculated above), we used the statistic described in the Supplementary Methods 1.7.1. RNA reads were pre-processed in an identical fashion to gDNA read data and rRNA sequences were removed using the SILVA database^78^ and SortMeRNA–v1.7^79^. We mapped non–rRNA reads to the Accumulibacter reference genome and to all ORFs in the metagenome assembly using Bowtie2^80^. For functional analysis of transcriptome analysis, we cross-referenced NCBI-annotations to KEGG gene identifiers. Due to current reorganisation of the NCBI Prokaryotic RefSeq Genome Re-annotation Project, we could not directly cross-reference current NCBI annotations to KEGG, therefore we cross-referenced Accumulibacter ORFs from the current analysis through a previous set of annotations made with RAPSearch2 (v2.10)^81^ and RefSeq NR (February 2013), with taxonomic assignments processed using MEGAN v4.70^77^ to obtain a consensus annotation for each ORF, using the lowest common ancestor method (LCA) using the following parameters: min support = 1, min score = 50, top percent = 10 and remaining parameters set to default. Annotated ORFs where subsequently annotated to KEGG pathways. We tested the presence of 16S rRNA FISH probes and previously defined polyphosphate kinase 1 (*ppk1*) PCR primers sequence as target sequences in our gDNA data using exact matching of probe or primer sequences to metagenome reads (Supplementary Table S2).

### Fluorescence *in situ* hybridisation

Samples fixed in paraformaldehyde were washed twice with 1% phosphate–buffered saline solution after 2 h and stored at −20 °C in a 50:50 mixture of 1% phosphate-buffered saline solution and 100% ethanol. Target organisms were analysed using EUBmix targeting most Bacteria (a mixture of EUB338, EUB338II and EUB338III)^82^. Specific oligonucleotide probes included PAOmix (PAO651, PAO462 and PAO846) targeting Accumulibacter–PAOs^13^; ACC–I–444 and ACC–II–444 targeting Accumulibacter–PAOs Types I and II^83^; Tet1–266 (Type I), Tet2–174 and Tet2–892 (Type II) and Tet3–654 (Type III) targeting *Tetrasphaera* putative P–accumulating organisms^50^; GAOmix (GAOQ989 and GB G2) targeting Competibacter GAOs^27^,^84^; DEF2mix (DF988 and DF1020) targeting *D. vanus* related GAOs cluster II^29^; and DF1013 and DF1004 targeting phylotypes within cluster III *Defluviicoccus*^85^, indicated as putative GAOs^86^.

### Metabolic-model based analysis

The anaerobic stoichiometric calculations obtained from the lab–scale batch studies were analysed with three metabolic models (the Pereira model, the Yagci model and the Hesselmann model) that predict the anaerobic metabolism of PAO with acetate via glycolysis and TCA cycle (Supplementary Figure S3), using carbon fluxes and reducing power balances described in Zhou *et al.*^19^ and Pijuan *et al.*^42^. The differences between the three models are that the Pereira model^17^ predicts the anaerobic operation of the full TCA cycle, while the Yagci model^87^ predicts the formation of reducing power through the partial TCA cycle via the glyoxylate pathway, and the third model proposed by Hesselmann *et al.*^88^ suggests a split operation of the TCA cycle where acetyl–CoA is converted to propionyl–CoA through the oxidative pathway via oxaloacetate (forward direction) and/or the reductive (reverse direction) via succinyl–CoA. The solver optimisation function in Microsoft Excel was used to solve the parameters in the metabolic models as also described by Zhou *et al.*^19^. The standard error for the parameter estimation was determined using jackknife resampling.

## Additional Information

**How to cite this article**: Law, Y. *et al.* Integrative microbial community analysis reveals full-scale enhanced biological phosphorus removal under tropical conditions. *Sci. Rep.* **6**, 25719; doi: 10.1038/srep25719 (2016).

## Supplementary Material

Supplementary Information

Supplementary Data

## Figures and Tables

**Table 1:** OTUs differentially abundant between high and low aeration epochs.

**OTU identifier**^1^	**Difference**^2^	**F-statistic**^3^	**Raw P**^4^	**q**^5^	**Annotation**^6^
denovo886	−0.32	104.85	7.11E-06	1.36E-03	k__Bacteria; p__Proteobacteria; c__Betaproteobacteria
denovo474	−0.65	54.49	7.76E-05	7.44E-03	k__Bacteria; p__Bacteroidetes; c__[Saprospirae]; o__[Saprospirales]
denovo4523	0.33	40.11	2.25E-04	1.44E-02	k__Bacteria
denovo4670	0.32	29.01	6.57E-04	2.52E-02	k__Bacteria
denovo5306	0.44	29.64	6.13E-04	2.52E-02	k__Bacteria; p__Actinobacteria; c__Actinobacteria; o__Actinomycetales; f__; g__; s__
denovo464	0.30	23.31	1.31E-03	2.79E-02	k__Bacteria; p__Proteobacteria; c__Betaproteobacteria; o__Ellin6067; f__; g__; s__
denovo890	0.40	23.43	1.29E-03	2.79E-02	k__Bacteria; p__Proteobacteria; c__Gammaproteobacteria; o__Salinisphaerales; f__Competibacteraceae; g__; s__^7^
denovo2451	0.19	26.50	8.77E-04	2.79E-02	k__Bacteria; p__Proteobacteria; c__Gammaproteobacteria; o__Salinisphaerales; f__Competibacteraceae; g__; s__
denovo2882	−1.09	25.22	1.03E-03	2.79E-02	k__Bacteria; p__Acidobacteria; c__[Chloracidobacteria]; o__PK29; f__; g__; s__
denovo3239	0.21	19.86	2.12E-03	4.07E-02	k__Bacteria; p__Proteobacteria; c__Alphaproteobacteria; o__Rhizobiales; f__; g__; s__
denovo16	−0.33	17.19	3.23E-03	4.80E-02	k__Bacteria; p__Proteobacteria; c__Betaproteobacteria
denovo51	0.31	16.87	3.40E-03	4.80E-02	k__Bacteria; p__Actinobacteria; c__Actinobacteria; o__Actinomycetales; f__Mycobacteriaceae; g__Mycobacterium
denovo854	−0.20	16.83	3.43E-03	4.80E-02	k__Bacteria; p__Bacteroidetes; c__Cytophagia; o__Cytophagales; f__Cytophagaceae; g__; s__
denovo2139	0.33	16.70	3.50E-03	4.80E-02	k__Bacteria; p__Chloroflexi; c__Anaerolineae; o__Caldilineales; f__Caldilineaceae; g__Caldilinea; s__
denovo3503	0.35	15.58	4.25E-03	4.86E-02	k__Bacteria; p__Proteobacteria; c__Betaproteobacteria; o__Rhodocyclales; f__Rhodocyclaceae; g__Candidatus Accumulibacter; s__
denovo3915	0.37	15.50	4.31E-03	4.86E-02	k__Bacteria; p__Proteobacteria; c__Gammaproteobacteria; o__Salinisphaerales; f__Competibacteraceae; g__; s__
denovo4686	0.33	16.16	3.84E-03	4.86E-02	k__Bacteria; p__Proteobacteria; c__Deltaproteobacteria; o__Myxococcales; f__Nannocystaceae; g__Nannocystis
denovo5418	−0.18	14.65	5.03E-03	5.36E-02	k__Bacteria; p__Bacteroidetes; c__[Saprospirae]
denovo2368	0.26	13.59	6.17E-03	6.23E-02	k__Bacteria; p__Proteobacteria; c__Gammaproteobacteria; o__Salinisphaerales; f__Competibacteraceae; g__; s__
denovo1196	0.38	12.90	7.07E-03	6.47E-02	k__Bacteria; p__Proteobacteria; c__Betaproteobacteria; o__Rhodocyclales; f__Rhodocyclaceae; g__Candidatus Accumulibacter; s__
denovo4321	0.43	12.89	7.08E-03	6.47E-02	k__Bacteria; p__Actinobacteria; c__Actinobacteria; o__Actinomycetales
denovo1045	−0.49	11.11	1.03E-02	7.91E-02	k__Bacteria; p__Bacteroidetes; c__[Saprospirae]
denovo2808	0.36	11.13	1.03E-02	7.91E-02	k__Bacteria; p__Proteobacteria; c__Betaproteobacteria; o__Burkholderiales; f__Comamonadaceae; g__Methylibium; s__
denovo2874	−0.41	10.78	1.11E-02	7.91E-02	k__Bacteria
denovo3702	0.27	10.82	1.10E-02	7.91E-02	k__Bacteria; p__Proteobacteria; c__Betaproteobacteria; o__Burkholderiales; f__Comamonadaceae; g__Roseateles; s__depolymerans
denovo3949	0.12	10.85	1.10E-02	7.91E-02	k__Bacteria; p__Proteobacteria; c__Alphaproteobacteria; o__Rhizobiales; f__; g__; s__
denovo5370	0.21	11.18	1.02E-02	7.91E-02	k__Bacteria; p__Actinobacteria; c__Actinobacteria; o__Actinomycetales; f__Nocardioidaceae; g__; s__
denovo56	0.58	10.08	1.31E-02	8.98E-02	k__Bacteria; p__Bacteroidetes
denovo1915	−0.27	9.75	1.42E-02	9.37E-02	k__Bacteria; p__Bacteroidetes
denovo843	0.41	9.20	1.63E-02	9.62E-02	k__Bacteria; p__Proteobacteria; c__Betaproteobacteria
denovo3134	−0.36	9.14	1.65E-02	9.62E-02	k__Bacteria; p__SR1; c__; o__; f__; g__; s__
denovo5082	−0.21	9.01	1.70E-02	9.62E-02	k__Bacteria; p__Chloroflexi; c__Chloroflexi; o__[Roseiflexales]; f__[Kouleothrixaceae]; g__Kouleothrix; s__
denovo5114	−0.21	9.14	1.65E-02	9.62E-02	k__Bacteria
denovo5130	0.25	9.05	1.69E-02	9.62E-02	k__Bacteria; p__Proteobacteria; c__Betaproteobacteria; o__Rhodocyclales; f__Rhodocyclaceae; g__Candidatus Accumulibacter; s__
denovo4776	0.18	8.80	1.79E-02	9.84E-02	k__Bacteria; p__Proteobacteria; c__Alphaproteobacteria; o__Rhodospirillales
denovo2836	−0.43	8.57	1.91E-02	9.89E-02	k__Bacteria; p__Bacteroidetes
denovo3464	0.32	8.65	1.87E-02	9.89E-02	k__Bacteria; p__Chloroflexi; c__Anaerolineae; o__Caldilineales; f__Caldilineaceae; g__Caldilinea; s__

^1^Internal identifier for operational taxonomic units (OTUs) generated from analysis of 16S rRNA gene amplicon sequencing reads.

^2^Difference in log_10_ normalised abundance between high and low-aeration epochs.

^3^Value of test statistic from change-point model (Reeve *et al.*^69^).

^4^Estimated (unadjusted) p-value.

^5^Significance feature estimated using Storey-Tibshirani false discovery rate estimator.

^6^Taxonomic assignment for OTUs was made from Greengenes or MIDAS.

^7^The annotation to “k__Bacteria; p__Proteobacteria; c__Gammaproteobacteria; o__Salinisphaerales; f__Competibacteraceae; g__; s__ ” was provided by MIDAS; all other annotations are from Greengenes.

**Table 2:** Comparison of the aerobic and anaerobic biochemical transformation with metabolic model predictions during high and low aeration epochs.

**Experimental date ormodel used**	**Ratio of key constituents**^1^						
	**Anaerobic**					**Aerobic**	
	**P/Hac**	**Gly/HAc**	**PHB/Hac**	**PHV/Hac**	**PHA/Hac**	**P/PHA**	**Gly/PHA**
Experimental							
High Aeration Period							
Date							
29th May	0.92	0.74	0.51	0.06	0.57	0.67	0.67
5th June	0.80	0.63	0.69	0.07	0.76	0.90	0.52
12th June	1.01	0.58	0.84	0.13	0.97	0.73	0.60
19th June	0.83	0.44	1.30	0.20	1.50	0.73	0.64
Mean of period (s.d.)	0.89 (0.09)	0.60 (0.12)	0.83 (0.34)	0.11 (0.06)	0.95 (0.40)	0.76 (0.10)	0.61 (0.07)
Low Aeration Period							
26th June	0.82	0.34	0.94	0.17	1.11	0.82	—
3rd July	0.99	0.39	0.96	0.20	1.16	0.84	0.53
10th July	0.86	0.34	1.03	0.18	1.21	0.72	0.49
16th July	0.93	0.37	1.01	0.21	1.22	0.75	0.51
25th July	0.94	0.44	0.90	0.19	1.09	1.04	0.47
14th August	0.81	0.38	0.81	0.19	1.00	0.76	0.65
Mean of period (s.d.)	0.89 (0.07)	0.38 (0.04)	0.94 (0.08)	0.19 (0.01)	1.13 (0.08)	0.82 (0.12)	0.56 (0.09)
Model							
Comeau *et al.*^15^ (Anaerobic PAO TCA model)	0.5	0	0.9	0	0.9	N/A	N/A
Smolder *et al.* (1994) (Anaerobic PAO Gly Model)	0.5	0.5	1.33	0	1.33	N/A	N/A
Pereira *et al.*^17^ (Anaerobic PAO TCA + Gly Model)	0.16	0.7	1.02	0.46	1.48	N/A	N/A
Hesselmann *et al.*^88^ (Anaerobic PAO partial TCA + Gly Model)	0.37	0.6	1.11	0.29	1.4	N/A	N/A
Yagci *et al.*^87^ (Anaerobic PAO partial TCA + Gly Model)^2^	*αPAO* + *1/2*(*1-1/*(*2* + *f*_*GLY*_))	*1/*(*2* + *f*_*GLY*_)	*8/*(*6* + *3f*_*GLY*_)	*2.5/*(*6* + *3f*_*GLY*_)	N/A	N/A	N/A
Smolder *et al.* (1994) (Aerobic PAO model)	N/A	N/A	N/A	N/A	N/A	0.41	0.42
Zeng *et al.* (2003) (GAO Model)	0.00	1.12	1.36	0.46	1.86	0.00	0.65

^1^P/HAc, phosphorus/acetate; Gly/HAc, glycogen/acetate; PHB/HAc, poly–hydroxybutyrate/acetate; PHV/HAc, poly-hydroxyvalerate/acetate; PHA/HAc, poly–β–hydroxyalkanoates/acetate; P/PHA, phosphorus/ poly–β–hydroxyalkanoates; Gly/PHA, glycogen/poly–β–hydroxyalkanoates.

^2^αPAO is the energy required to transport one C mole of acetate across the cell membrane. f_GLY_ is the fraction of acetyl-CoA going through the glycoxylate pathway to produce reducing power. Values are in C or P mole.
