# Supplementary material for: Integrative microbial community analysis reveals full-scale enhanced biological phosphorus removal under tropical conditions
Source: Sci Rep. 2016 May 19;6:25719. doi: 10.1038/srep25719 (PMC4872125; doi:10.1038/srep25719)
Supplement: Supplementary Information [file srep25719-s1.pdf]

Supplementary Methods, Results, Tables and Figures for *Integrative microbial community analysis reveals full-scale enhanced biological phosphorus removal under tropical conditions*

Yingyu Law, Rasmus H. Kirkegaard, Angel Anisa Cokro, Xianghui Liu, Krithika Arumugam, Chao Xie, Mikkel Stokholm–Bjerregaard, Daniela I. Drautz-Moses, Per H. Nielsen, Stefan Wuertz, Rohan B.H. Williams

Contents

1 Supplementary Methods **2**
- 1.1 DNA extraction . . . 2
- 1.2 RNA extraction . . . 3
- 1.3 16S amplicon sequencing . . . 4
- 1.4 Metagenome sequencing . . . 4
- 1.5 Metatranscriptome sequencing . . . 4
- 1.6 Analysis of community composition and dynamics . . . 5
- 1.7 Metagenome and comparative genome analysis . . . 6
  - 1.7.1 ORF–level descriptive statistics for contigs and bins . . . 6
  - 1.7.2 Differential coverage estimation . . . 6

2 Supplementary Results **7**
- 2.1 Additional comments on FISH probe in metagenome data . . . 7
- 2.2 Metagenome assembly statistics . . . 7
- 2.3 Simulations for assessing power of test statistics . . . 7
- 2.4 Influence of temporal correlation on test statistics . . . 8
- 2.5 Comparative analysis of pathway expression in Accumulibacter . . . 9
- 2.6 Are changes in the Accumulibacter transcriptome consistent with changes in Accumulibacter abundance? . . . 10

List of Tables

S1 eFISH probe abundance in metagenome data 11
S2 Metabolic model predictions for high– and low–aeration epochs 12
S3 Metagenome co–assembly statistics 13
S4 Comparative analysis against extant Accumulibacter genomes 14
S5 Pathway level expression data in Accumulibacter 15
S6 Operational data for Ulu Pandan Water Reclamation Plant 16

List of Figures

S1 Phosphate transformation data from field sampling 17
S2 Additional results on signal detection for 16S data 18
S3 Schematic diagrams for anaerobic metabolism pathways in PAOs 19
S4 Amino acid identity with respect to extant Accumulibacter reference and draft genomes 20
S5 Differential abundance of contigs between high– and low–oxygenation epochs 21
S6 Analysis of expression–differences in Accumulibacter genes, between high– and low–aeration epochs 22
S7 Schematic diagram of Ulu Pandan Water Reclamation Plant (South Works) with sampling locations highlighted 23
S8 Signal detection simulations related to 16S data 24
S9 Analysis of influence of autocorrelation on false positive rate (16S data) . . 25

1 Supplementary Methods

1.1 DNA extraction

The DNA was extracted based on the FastDNA^{TM} 2 mL SPIN Kit for Soil (MP Biomedicals, USA) optimised for DNA extraction from activated sludge by one of us (P.H.N.) and co–workers. The sludge sample was defrosted and subsequently homogenised with a Heidolph RZR 2020 overhead stirrer (Heidolph Instruments, DE) for 1 min at gearing II speed 9. Subsequently, 1.0 mL of the homogenised sample was transferred to a 2 mL tube and centrifuged at 21,100 g for 5 minutes in a Sorvall Legend Micro 21 Microcentrifuge (Thermo Fischer Scientific, USA). The supernatant was discarded and the pellet was resuspended in 978 μL sodium phosphate buffer (pH 8). Resuspended cells were transferred to a Lysing matrix E tube and 122 μL MT buffer was added. The sample was then homogenised using a FastPrep FP120 Homogenizer (Thermo Savant, USA) for 4×40 seconds at speed 6, and the samples were stored on ice for 2 min between each bead beating. After this step the manufacturers protocol was followed.

1.2 RNA extraction

RNA extractions were performed with Zymo Research ZR Soil/ Fecal RNA Isolation Kit (Catalog no. R2040). Specifically, a 500 μL volume of frozen biomass was left to completely thaw on ice was then added to a 2.0 mL reaction tube and centrifuged at 12,000 rpm for 3 minutes. The pellet was then lysed by adding 1.0mL of S/F RNA Lysis Buffer and was kept on ice for 2 minutes to obtain improved yield. The mixture was added to the ZR Bashing Bead Lysis Tube and lysed by bead beating in a Fast Prep Instrument, at a speed of 6.0m/s for 40 seconds to extract total RNA. The lysate was centrifuged at 12000 rpm for 1 minute, then 400 μL of supernatant was transferred to a clean 1.5 mL RNAse–free tube and an equal volume of 400 μL RNA Binding Buffer was added. This mixture was transferred into a Zymo Spin IIIC column in a collection tube and was centrifuged at 3000 rpm for 30 seconds. 800 μL of 100% ethanol was added and mixed well to precipitate RNA. The mixture was again transferred to a new Zymo Spin IIIC column in a collection tube which was then centrifuged twice at 12000 rpm for 30 seconds. The column was transferred into a clean RNAse–free tube and 100 μL DNase/RNAse–free water was added directly to the column matrix and was left to stand at room temperature for 2 minutes. After which it was centrifuged at 12000 rpm for 30 seconds. The eluted RNA was transferred into a Zymo Spin IV–HRC Spin Filter in an RNAse free tube and was centrifuged at 8000 rpm for 1 minute to remove any RT–PCR inhibitors (*e.g.* humic acids, polyphenols, tannins). 100 μL RNA Binding Buffer and 100 μL 100% ethanol were added to the filtrate and mixed well by pipetting. The mixture was transferred into a Zymo Spin IC Column in a collection tube and was centrifuged at 12000 rpm for 1 minute. The flow through was discarded and 400 μL RNA Prep Buffer was added to the column and centrifuged at 12000 rpm for 1 minute. The supernatant was discarded and 700 μL RNA Wash Buffer was added and was centrifuged at 12000 rpm for 30 seconds. The supernatant was removed, followed by a final wash of 400 μL RNA Wash Buffer and was centrifuged at 12000 rpm for 30 seconds. The column was emptied and it was centrifuged again at 12000 rpm for 2 minutes to ensure complete removal of wash buffer. The column was placed carefully into an RNase–free tube and 16 μL of DNase/RNase–Free Water was added directly to the column matrix and was incubated for 2 minutes at room temperature. The sample was centrifuged at 10000 rpm for 30 seconds. A second elution was done to obtain a better yield of concentrated purified RNA. However, complete removal of DNA was necessary and was achieved by performing a DNase digestion with the help of Turbo DNAse Digestion Kit (Catalog Number AM 1907). The DNA digested RNA extract was then further purified by RNA Clean and Concentrator–5 (Catalog no. R1016). The procedures entailed a single–buffer system and Fast-Spin column technologies: 100 L RNA Binding Buffer and 100 μL 100% ethanol was added to the sample to allow binding onto the specially formulated column matrix. The RNA was then washed with 800 μL RNA Wash Buffer followed by a 400 μL Wash Buffer. 16 μL RNAse Free Water was used to elute concentrated RNA and a second elution step was repeated for the maximum recovery of total RNA. Aliquots of

RNA extract were prepared in PCR tubes to minimise RNA degradation and were stored at −80 °C.

1.3 16S amplicon sequencing

The bacterial primers used were 27F (AGAGTTTGATCCTGGCTCAG, Lane 1991) and 534R (ATTACCGCGGCTGCTGG, Muyzer et al, 1993), which amplifies a DNA fragment of 500 bp of the 16S rRNA gene (Variable V1 to V3 region). PCR amplification was performed using 1X Platinum® High Fidelity buffer, 400 pM dNTP, 1.5 mM $MgSO_4$, 2 mU Platinum® Taq DNA Polymerase High Fidelity, 5 μM barcoded V1–V3 adaptor mix, and 10 ng template DNA. PCR conditions were 95 °C, for 2 min, 30 cycles of 95 °C, for 20 sec, 56 °C for 30 sec, 72 °C for 60 sec and then a final step of elongation at 72 °C for 5 min. PCR products were purified using Agencourt AmpureXP (Beckman Coultier) with a ratio of 1.8 bead solution/PCR solution. The concentration of DNA was determined using the QuantIT HS kit (Life Technologies). Barcoded amplicons were pooled in equimolar amounts and paired–end sequenced (2×250 bp) on an Illumina MiSeq (Illumina Inc).

1.4 Metagenome sequencing

Prior to library preparation, the quality of the DNA samples was assessed on a Bioanalyzer 2100, using a DNA 12000 Chip (Agilent). Sample quantitation was performed using the Picogreen assay (Invitrogen). Next–generation sequencing library preparation was performed by following the Illumina TruSeq DNA Sample Preparation protocol with the following modifications: For each sample, 1 μg of the DNA was sheared on a Covaris S220 to approximately 300bp, following the manufacturers recommendation. Size selection was performed on a Sage Science Pippin Prep instrument, using a 2% EtBr agarose cassette and selecting for a tight peak around 400 bp. Each library was tagged with a TruSeq LT DNA barcode (Illumina) to allow for library pooling prior to sequencing. Library quantitation was performed using the Picogreen assay (Invitrogen) and the average library size was determined by running the libraries on a Bioanalyzer DNA 7500 chip (Agilent). Library concentrations were normalized to 4 nM and validated by qPCR on a ViiA–7 real-time thermocycler (Applied Biosystems), using qPCR primers recommended in Illumina in their qPCR protocol, and the Illumina PhiX control library was used as a standard. Libraries were then combined in one pool, which was sequenced across two lanes of an Illumina HiSeq2500 sequencing run at a read–length of 151 bp paired–end.

1.5 Metatranscriptome sequencing

Prior to library preparation, the quality of the RNA samples was determined by running all samples on a Bioanalyzer RNA 6000 Nano Chip (Agilent). Sample quantitation was performed using the Invitrogen Ribogreen assay (Thermo Fisher Scientific), and to rule out DNA contamination, RNA samples were also subjected to the Invitrogen Picogreen assay.

Next–generation sequencing library preparation was performed by following the Illumina TruSeq Stranded mRNA protocol (Illumina) with the following modifications: the mRNA purification step in both protocols was omitted and instead, 200 ng of total RNA were directly added to the elute–fragment–prime step. The PCR amplification step, which selectively enriches for library fragments that have adapters ligated on both ends, was performed according to manufacturer recommendation but the number of amplification cycles was reduced to 12. Each library was uniquely tagged with one of the Illumina TruSeq LT RNA barcodes to allow library pooling for sequencing. Library quantitation was performed using the Invitrogen Picogreen assay and the average library size was determined by running the libraries on a Bioanalyzer DNA 1000 chip (Agilent). Library concentration was normalized to 2nM and the concentration was validated by qPCR on a ViiA–7 real-time thermocycler (Applied Biosystems), using qPCR primers recommended in the Illumina qPCR protocol and the Illumina PhiX control library as a standard. The libraries were pooled at equal volumes and sequenced in two lanes of an Illumina HiSeq2500 rapid run at a final concentration of 9pM and a read–length of 101bp paired–end.

1.6 Analysis of community composition and dynamics

The relative abundance of each OTU was estimated by summing the total number of reads associated reads and expressing this number a quotient to the total number of reads. Relative abundances were summed across multiple OTUs annotated to the same taxa. To test differences between the two epochs with high and low levels of aeration, we used a test statistic designed to measure step–like changes in the mean level of time series data (Reeves *et al.*, 2007), first conforming using simulation that we had adequate power using only 10 samples split into groups of 4 and 6 (see **Fig. S8**). Specifically, for a time series R_n with samples indexed by $n = 1, \ldots, N$, we tested a hypothesis that a shift in mean level will occur between samples 1–4 and 5–10 using the following test statistic:

$$F_k = \frac{SSE_O - SSE_A}{\frac{SSE_A}{N-3}} \tag{1}$$

where:

$$SSE_O = \sum_{j=1}^{k} (R_j - \hat{R}_{(1,k)})^2 + \sum_{j=k+1}^{N} (R_j - \hat{R}_{(k+1,N)})^2 \tag{2}$$

$$SSE_A = \sum_{j=1}^{N} (R_j - \hat{R}_{(1,N)})^2 \tag{3}$$

and:

$$\hat{A}_{(a,b)} = \frac{\sum_{j=a}^{b} R_j}{b - a + 1} \tag{4}$$

we computed this test statistic for each included taxa, and the estimated P–values from the F–distribution, $F(m,k)$ with $m = 1$ and $k = 8$, and corrected these for the number of tests conducted using the Storey–Tibshirani estimator (Storey and Tibshirani, 2003), after confirming that distribution of unadjusted P–values was consistent with the application of this procedure (**Fig. S2A**). We checked the abundance residuals for first–order autocorrelation and inhomogeneities of variance that may have resulted in an increased false positive rate (see **Supplementary Results 2.4** below).

1.7 Metagenome and comparative genome analysis

Further methods not described in the main text are provided here.

1.7.1 ORF–level descriptive statistics for contigs and bins

We used a simple descriptive statistic that could quantity the extent to which ORF–level annotations to the same taxonomic entity occurred acoss contigs and bins defined by MetaBAT. Starting with the `lcamapper` output data, for a given ORF O, and a given taxonomic assignment, A (here, mostly at genus level) we define a indicator variable, T^A that is set to one if 100% of supra–threshold taxonomic assignments are made to A and zero otherwise. Thus, this statistic captures ORFs that have highly consistent annotations to the taxon of interest. If ORF $i = 1, \ldots N$ resides on contig $j = 1, \ldots, M$, which is a member of bin k, and T_{ij} is the corresponding indicator score, then the summary statistic for the entire, $S_k(A)$, is:

$$S_k(A) = \frac{1}{NM} \sum_j \sum_i T_{ij} \tag{5}$$

see **Supplementary Table 5**, columns HERE for examples of this statistic calculated for genus Accumulibacter and related genera from *Rhodocyclaceae*)

1.7.2 Differential coverage estimation

To avoid re–using data that were used in constructing the assembly for the estimation of coverage profiles, we used a replicated gDNA survey from duplicate samples from each of the 10 sampling events (using identical protocols to that described above) to estimate coverage matrix from the co–assembly. Following removal of contigs that were not detectable in all 10 samples, we applied a log base 10 transformation and further removed inter–sample variation using quantile normalization. To calculate differential contig abundance between high– and low–oxygenation epochs, we generated a contig–by-sample coverage matrix, $C = [c_{ij}]$, where c_{ij} is the normalised coverage of contig i in sample j, from the independent replication of the samples. Differential abundance for i–th contig was calculated as the difference between normalised abundance (log_{10}) in the high–oxygenation epoch, $C_{(i,high)}$,

to the normalised abundance in the low–oxygenation epoch, $C_{(i,low)}$ or $\Delta_i = C_{(i,high)} - C_{(i,low)}$. See **Fig. S5**.

2 Supplementary Results

2.1 Additional comments on FISH probe in metagenome data

In addition to the 16S FISH probes sourced from Nielsen, Daims and Lemmer (2009), we also tested for the presence of reveal previously defined *polyphosphate kinase 1* (ppk1) probe sequences in our gDNA data (refer to **Table S1** for details of probe sequences detected in all 10 samples). Other relevant observations are as follows. Clade IIA was detected in all 10 samples using the Acc–II–444 probe, but was only partially detected (4/10 samples), and at low levels (maximum 2 read counts), using the Acc–ppk1–997r probe and not detected at all using the Acc–ppk1–893f probe sequence. Clade IIB was present in all 10 samples (Acc–IIB–ppk1 and Acc–ppk1–1002r). Clade IIC was identified in all samples using multiple probes (Acc–IIC–ppk1 and Acc–ppk1–460r), albeit at different relative abundances. Neither probe targeting Clade IID (Acc–IID–ppk1 and Acc–ppk1–522r) were not identified in any sample. Clade IIF was partially detected in both probes used (AcceIIF–ppk1 and Acceppk1–600r; 3/10 samples, with a maximum of 2 read counts detected). Clade I was detected in all samples, at low levels, using two probes, and partially detected using a third (Acc-I-444; detected in 5/10 samples, with a maximum read count of 2).

2.2 Metagenome assembly statistics

Assembly statistics are reported in **Table S3**. Briefly, the metagenome co–assembly generated from gDNA read sequenced from all 10 sampling events and was comprised of 2,667,112 (minimum contig length of 500bp; 804,992 (30.2%) were greater than length 1000bp; see **Table S3**). From the predicted set of 4,592,053 genes residing on these contigs, we could identify 34165 that held consensus annotations to Accumulibacter under the MEGAN–LCA algorithm. These ORFs were distributed across 22967 contigs (see Methods), of which 8380 contained a single ORF, and 4274 had multiple ORF that were all consistently annotated to the taxon. We can observe at least one instance of 3937 of the 4562 coding genes in the reference genome (86%), or 3352 (73%) if we only considered ORFs from the consistently annotated and singleton contigs.

2.3 Simulations for assessing power of test statistics

To establish whether we had sufficient power to detect the differences in abundance between high– and low–oxygenation epochs using the change–point statistics with a total of 10 samples, we undertook a prospective analysis of statistical power using simulations. These

are by nature non–exhaustive, but offer some level of insight into the behaviour of the test statistics used here, under conditions close to those of the actual data. We constructed simulated from the observed OTU abundance matrices as outlined below, and undertook the following analyses from these simulated data:

1. We first tested whether the false positive rate was well controlled by generating an ensemble of 1000 of 337×10 matrices, resampled with replacement from the double–centered abundance (observed) matrix, and tested for differential abundance using identical methods to those used on the observed data. We also generated a second set of null data, using the same method but following removal of the influence of the first principal component, in order to further remove any residual variation associated with change in abundance over time. Analysis with both these null sets confirmed the that the false positive rate was well controlled under the conditions of the study (**Fig. S8A**).

2. We next performed analyses to examine our ability to detect signal under the specific conditions similar to those in the observed data. Using the ensemble of randomly selected a subsets of OTUs and introduced a random, non–zero change in abundance between high– (samples 1–4) and low–oxygenation (samples 5–10) epochs, in a subset of rows. We modelled the abundance offset using several different distributions, and set the number of OTUs that were differentially abundant to 20, 50 and 100, respectively. From each of these sets of simulated data, we performed a differential abundance using identical methods to those employed in the observed data, and classified the results using receiver operator characteristic (ROC) analysis, across a range of values of the test statistic F (see legend to **Fig. S8B** for further details).

2.4 Influence of temporal correlation on test statistics

Temporal correlation long been recognised to be the major factor that potentially increase false positive rate when performing statistical hypothesis testing on time series data (Millard *et al.*, 2009). We therefore examined whether there was evidence for our test statistics being subject to this effect using established methods (Millard *et al.* 2009 and De Carlo and Tryon 1993. Specifically, from the time series data, R_n, with $n = 1, \dots, N$, from a given 16S OTU marker, we study the residuals of this series following removal of mean effects from both high– and low–oxygenation epochs, namely U_n for $n = 1, \dots, N$ and compute the first order autocorrelation from them as:

$$r_1 = \frac{\sum_{n=1}^{N-1}(U_n - \hat{U})(U_{n+1} - \hat{U})}{\sum_{n=1}^{N-1}(U_n - \hat{U})} \tag{6}$$

where here, we have $N = 10$. We first confirmed that this distribution of is negatively biased by an amount approximately $1/N$, as expected (De Carlo and Tryon, 1993), and so

instead use a bias–reducing estimator, r_1+, calculated as:

$$r_1+ = r_1 + \frac{1}{N} \tag{7}$$

which removed the bias evident in r_1 (**Fig S9A**). Examining the relationship between the degree of autocorrelation and the significance features used in our analysis (q–values), we see no evidence for systematic bias across the range of autocorrelation observed (**Fig. S9B**). Finally, we formally tested whether there was any evidence for increased levels of autocorrelation in the set of 16S OTU detectable in all 10 samples and which are the focus of our analysis. For the r_1+ estimator, the following statistic:

$$T = \frac{r_1+}{\left(\frac{n-2}{n\sqrt{n-1}}\right)}$$

will be normally distributed. In **Fig. S9C** we show histograms of P–values for both the left– and right–tail comparisons (which test for negative and positive autocorrelation, respectively), as well as a $Q-Q$ plot of T against the normal distribution in **Fig. S9D**. We can observe that the empirical T follows the normal distribution well, except for the extremes of both tails. Collectively then, there is little evidence for an increase in autocorrelation, and we can conclude that temporal correlation has not increased the bulk false positive rate in the analysis of differential abundance.

2.5 Comparative analysis of pathway expression in Accumulibacter

Using gene–referenced to the Accumulibacter reference genome, we considered whether expression of genes in the TCA pathway (KEGG `00020`) were stochastically higher those in the Glycolysis pathway (KEGG `00010`). This analysis is complicated by the shared, therefore, we undertook three variants on this analysis, testing the difference between the two distributions using unpaired $t-$test (due to the presence of ties in the data, due to effect of missing values, we could not use the preferred option of the non–parametric Mann–Whitney U test). We performed the following analyses *(1)*: we tested all genes in the TCA pathway against those in the Glycolysis pathway that were not members of the former pathway; *(2)*: we tested all genes in the TCA pathway that were not members of the Glycolysis pathway against all members of the later, and *(3)*: we tested members of the TCA pathway against members of the Glycolysis pathway, excluding from both groups, any gene that was a member of the other group. The results of this analysis of difference in mean (log2) expression (TCA vs. Glycolysis) were as follows: *(1)* 3.37 vs. 2.41, $t = 1.96$, $P = 0.028$ *(2)* 3.29 vs. 2.72, $t = 1.11$, $P = 0.138$ and *(3)* 3.29 vs. 2.72, $t = 1.57$, $P = 0.063$. Therefore we conclude there is some statistical support for increased expression in the TCA pathway compared to the Glycolysis pathway, but this is of only moderate significant and is highly related to the degree of overlap among their member genes.

2.6 Are changes in the Accumulibacter transcriptome consistent with changes in Accumulibacter abundance?

We sought to determine whether changes in gene expression observed in genes annotated to Accumulibacter where consistent in changes in abundance between the high– and low–aeration epochs. While the differential expression analysis in principal contains this information, the fact that ORFs from all taxa are included in the analysis implies that the changes in the taxon of interest will be enriched relative to all others (in order to be detectable) . In biological terms, this assumption appears overly restrictive and unrealistic, as we could in visage a taxon in which the overall transcriptome state remains the constant, but by virtue of an increase in taxon abundance, will be observed to increase its expression levels. Therefore addressing this question requires a more targeted approach. To do so, we first took all genes referenced to Accumulibacter, and constructed a reference distribution for their change in expression between the high– and low–aeration epochs. The reference distribution will describe what expected changes in expression are observable and therefore we can determine whether the changes in our taxon of interest are different from expectation. Specifically, for a given gene g for which N_g ORFs are annotated, we will have a set of ORFs $O_g = \{o_{(g,1)}, o_{(g,2)}, \ldots, o_{(g,N_g)}\}$ with missing value counts $M_g = \{m_{(g,1)}, m_{(g,2)}, \ldots, m_{(g,N_g)}\}$. If Δ_k is the difference in normalised read count (log_2 scale) for the k–th ORF, then our primary statistic of interest is the mean difference across the set of ORF O_g, namely $S_g = N_g^{-1} \sum_{j=1}^{N_g} \Delta_{o_{(g,j)}}$. We then construct B randomised versions of this statistic namely $\{S_g^*\}$, preserving the number of component ORFs and missing values counts, by conditional sampling with replacement from the full set of G ORFs. We compute standardised statistics, $Z_g = \frac{S_g^* - \mu_g}{\sigma_g}$ where $\mu_g = B^{-1} \sum_{j=1}^{B} S_g^*$ and $\sigma_g = \sqrt{(B-1)^{-1} \sum_{j=1}^{B} (S_j^* - \mu_g)^2}$. Under the analysis, we predict to see an *increase* in expression levels of genes annotated to Accumulibacter, relative to other genes, which will be supported by positive values of standardised statistic, Z_g. See **Fig. S6**.

Table S1: eFISH probe abundance[a] in metagenome data (normalised to EUB338 count). Probes sequence and annotations taken from Nielsen, Daims and Lemmer (2009).

ProbeName	ProbeType	ProbeDescription	Mean % (s.d.)
TM7905	General	Most division TM7	0.68 (0.39)
Pla46	General	Planctomycetales	2.65 (0.61)
Myc657	General	Mycobacterium sub division Mycolata	1.40 (0.63)
MNP1	General	Mycolata	0.83 (0.43)
LGC354C	General	Firmicutes	0.65 (0.23)
HGC1156	General	Actinobacteria	6.16 (3.23)
GNSB941	General	Phylum Chloroflexi	5.43 (1.63)
GAM42a	General	class Gammaproteobacteria	8.98 (3.18)
EUB338-III	General	Verrucomicrobiales	2.96 (0.63)
EUB338-II	General	Planctomycetales	2.24 (0.46)
EUB338	General	Most Bacteria	100.68 (12.36)
CMN119	General	Suborder CMN exluding Dietzia spp and Tsukamurellla spp	1.41 (0.72)
CFX1223	General	Phylum Chloroflexi	5.29 (1.35)
CFB719	General	Most members of Bacteroidetes	6.67 (1.45)
CFB563	General	Most Flavobacteria	0.48 (0.16)
CFB286	General	Most of genus Tannerella and genus Prevotella of class Bacteroidetes	0.21 (0.14)
CF319ab	General	Most Flavobacteria	2.58 (0.63)
BET42a	General	class Betaproteobacteria	18.38 (3.47)
ALF968	General	class Alphaproteobacteria	13.17 (2.55)
Nso1225	AOB	Betaproteobacterial ammonia-oxidizing bacteria	0.88 (0.20)
Nsm156	AOB	Nitrosomonas spp, Nitrosococcus mobilis	0.24 (0.18)
TM7305	Filamentous		0.48 (0.26)
Strept	Filamentous	Streptococcaceae	0.56 (0.25)
SNA	Filamentous	Sphaerotilus natans	0.32 (0.21)
SAP-309	Filamentous	Most of Saprospiraceae	4.11 (1.35)
Noli-644	Filamentous		0.22 (0.12)
LDI	Filamentous	Leptothrix discophora	1.35 (0.59)
HHY-654	Filamentous	H. hydrossis and Isolate 10B	0.54 (0.27)
HHY	Filamentous	H. hydrossis	0.53 (0.28)
CHL1851	Filamentous		0.41 (0.23)
CFX109	Filamentous		0.74 (0.41)
ACA23a	Filamentous	Acinetobacter spp.	0.17 (0.10)
GB_G2	GAO	Some Competibacter	0.27 (0.23)
GB_G1	GAO	Some Competibacter	0.58 (0.33)
GB	GAO	Most Competibacter	0.56 (0.34)
GAOQ989	GAO	Some Competibacter	0.58 (0.33)
GAOQ431	GAO	Some Competibacter	0.47 (0.33)
Gam1278	GAO	Some Gammaproteobacteria	0.42 (0.32)
RHC439	PAO	Rhodocyclus or Accumulibacter	0.36 (0.13)
RHC175a	PAO	Most Rhodocyclaceae	5.81 (1.41)
PAO846b	PAO	Rhodocyclus tenuis group	6.02 (1.63)
PAO846	PAO	Most Accumulibacter	5.09 (1.52)
PAO651	PAO	Most Accumulibacter	3.41 (1.13)
HGC69a	PAO	Actinobacteria - high GC gram positive	4.01 (2.06)
Acc-II-444	PAO	Clade IIA	3.16 (1.33)
Acc-IIB-ppk1	PAO	Clade IIB	0.48 (0.26)
Acc-IIC-ppk1	PAO	Clade IIC	1.50 (0.41)
Acc-ppk1-763f	PAO	Clade I	0.22 (0.20)
Acc-ppk1-1170r	PAO	Clade I	0.23 (0.15)
Acc-ppk1-1002r	PAO	Clade IIB	0.13 (0.09)
Acc-ppk1-460r	PAO	Clade IIC	0.27 (0.13)
Ntspa712	NOB	Phylum Nitrospirae	1.36 (0.64)
Ntspa662	NOB	Genus Nitrospira	1.46 (0.63)
Ntspa1431	NOB	Nitrospira sublineage I	1.72 (0.73)
Ntspa1026	NOB	Nitrospira sublineages I and II	1.46 (0.80)

[a]We only included FISH probes that are consistently detected in all samples.

Table S2: Experimental results and model predictions using the optimized model parameters fitted to the experimental data during high and low aeration epochs

High aeration epoch							
Data/model	α	β	γ	δ	Gly/HAc[a]	PHB/HAc	PHV/HAc
Experiment	–	–	–	–	0.60 ± 0.12	0.83 ± 0.34	0.11 ± 0.06
Pereira model	0.368 ± 0.033	0.086 ± 0.001	0.809 ± 0.020	–	0.60 ± 0.12	0.99 ± 0.05	0.30 ± 0.04
Hesselmann model	0.143 ± 0.014	0.042 ± 0.004	0.245 ± 0.055	-0.015 ± 0.001	0.60 ± 0.12	0.94 ± 0.13	0.66 ± 0.37
Yagci model	1.024 ± 0.011	-0.018 ± 0.008	–	–	0.60 ± 0.12	1.41 ± 0.06	0.055 ± 0.007
High aeration epoch							
Data/model	α	β	γ	δ	Gly/HAc[a]	PHB/HAc	PHV/HAc
Experiment	–	–	–	–	0.38 ± 0.04	0.94 ± 0.08	0.19 ± 0.01
Pereira model	0.392 ± 0.004	0.086 ± 0.001	0.816 ± 0.002	–	0.60 ± 0.12	0.99 ± 0.05	0.30 ± 0.04
Hesselmann model	0.230 ± 0.004	0.070 ± 0.001	0.148 ± 0.002	-0.557 ± 0.003	0.38 ± 0.04	0.93 ± 0.02	0.42 ± 0.02
Yagci model	0.913 ± 0.007	0.065 ± 0.002	–	–	0.38 ± 0.04	1.14 ± 0.02	0.088 ± 0.003

[a] The measured Gly/Hac ratios were used for parameter estimation.
Standard errors are provided for the optimised model parameters.
Standard deviations are provided for experimental data and predicted PHB/HAc and PHV/HAc.

Table S3: Metagenome co–assembly statistics

	Contigs	Scaffolds
N	2,667,112	2,686,265
Total assembly length (bp)	2,962,570,466	3,082,571,824
Mean sequence length (bp)	1,111	1,148
Median sequence length (bp)	751	761
Maximum sequence length (bp)	218,141	345,362
Minimum sequence length (bp)	500	500
N50 (bp)	1,179	1,241
Length > 500bp (N)	2,658,490	2,677,855
Length > 1kbp (N)	804,992	842,280
Length > 10kbp (N)	10,509	12,406
Length > 100kbp (N)	11	24

Table S4: ORF–level comparative analysis against extant Accumulibacter genomes

Genome	N_{ORF}	AAI (mean)	AAI (median)	N_{ORF} (top 5%)	%ORF (top 5%)	Clade
Ca. Accumulibacter	40248	73.69	75.43	2979	7.40	—
Ca. Accumulibacter phosphatis	21846	73.91	75.42	460	2.11	IIA
Ca. Accumulibacter sp. SK-01	3539	78.42	83.70	871	24.61	IIC
Ca. Accumulibacter sp. SK-02	2139	80.34	86.00	667	31.18	IIC
Ca. Accumulibacter sp. BA-91	4795	74.02	75.97	635	13.24	IIC
Ca. Accumulibacter sp. SK-11	1299	70.15	72.20	53	4.08	IIF
Ca. Accumulibacter sp. SK-12	1437	69.59	70.50	122	8.49	IIF
Ca. Accumulibacter sp. BA-94	1860	74.81	77.79	95	5.11	IIF
Ca. Accumulibacter sp. BA-93	1130	65.52	65.58	17	1.50	IA
Ca. Accumulibacter sp. BA-92	2202	64.64	64.90	59	2.68	IC

Genome: Ca. Accumulibacter refers to genus level annotations; *Ca.* Accumulibacter phosphatis refers to the IIA str. UW-1 reference genome (see ref. 38 main text); all remaining draft genome from ref. 23.
AAI: amino acid identity (%); *mean*, mean AAI across all included; *median*, median AAI across all included ORFS
N_{ORF}*(top5%)*: number of ORFs in top 5–th %–ile of AAI distribution
%ORF(top5%): percentage of ORFs in top 5–th %–ile of AAI distribution
Clade: known Type and Clade association of reference genome

Table S5: Metabolic pathways in Accumulibacter ranked by overall gene expression level (top 20)

KEGG ID	Genes[a] (N)	Present[b] (N)	Expressed[c] (N)	ORF[d] (N)	Read count (total)	Pathway
app00072	4	4	4	28.00	24.95	Synthesis and degradation of ketone bodies
app00310	10	9	9	84.00	19.68	Lysine degradation
app00380	12	9	9	88.00	16.47	Tryptophan metabolism
app00362	12	8	8	88.00	14.43	Benzoate degradation
app00640	38	35	32	298.00	13.97	Propanoate metabolism
app00650	35	32	31	309.00	13.68	Butanoate metabolism
app00020	32	30	28	215.00	13.38	Citrate cycle (TCA cycle)
app00680	30	24	21	216.00	11.82	Methane metabolism
app00730	9	9	8	45.00	11.80	Thiamine metabolism
app00010	38	34	29	330.00	11.57	Glycolysis / Gluconeogenesis
app00620	51	48	41	401.00	11.40	Pyruvate metabolism
app01200	114	100	91	831.00	9.68	Carbon metabolism
app00071	16	14	12	139.00	9.42	Fatty acid degradation
app00633	8	8	6	42.00	8.90	Nitrotoluene degradation
app00280	23	22	20	173.00	8.49	Valine, leucine and isoleucine degradation
app04122	13	13	13	65.00	8.39	Sulfur relay system
app00630	44	39	36	262.00	8.21	Glyoxylate and dicarboxylate metabolism
app00030	15	10	10	117.00	8.20	Pentose phosphate pathway
app00900	15	15	15	119.00	8.09	Terpenoid backbone biosynthesis
app00190	63	57	53	434.00	7.52	Oxidative phosphorylation

[a] Number of genes in the canonical KEGG pathway for Accumulibacter.
[b] Number of genes in the canonical KEGG pathway detected in metagenome assembly.
[c] Number of genes in the canonical KEGG pathway detected in metatranscriptome.
[d] Total number of ORFs annotated to any gene in this pathway.

Table S6: Operational parameter and process performance of Ulu Pandan Water Reclamation Plant

	Units	Value
Operational Parameters		
Average flow	10^3 m3/day	200 ± 7
	10^3 m3/day/tank	17 ± 2
Hydraulic Retention Time	h	13
Solid Retention Time	day	6
pH		6.7 ± 0.2
Temperature	°C	$30 - 31$
Mixed Liquor Suspended Solids	mg/L	1.4 ± 0.2
Supplementary Chemical Precipitation		None
Influent		
Average Total Kjehldahl nitrogen (TKN)	mg–N/L	48 ± 8
Average Ammonium	mg–N/L	34 ± 2
Average Total P	mg–P/L	8 ± 1
Average Total Chemical Oxygen Demand	mg/L	315 ± 61
Nutrient Removal Efficiency		
COD Removal	%	90
N Removal	%	81
P Removal	%	72

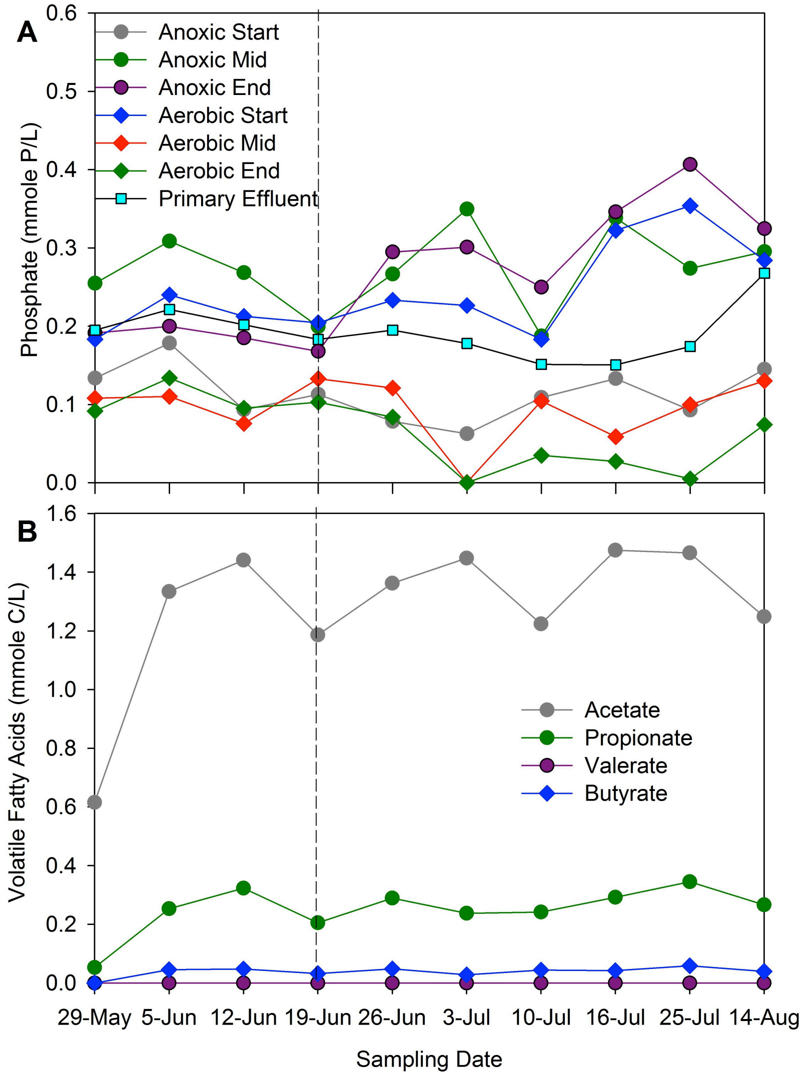

Figure S1: Changes in *(A)* phosphate transformation activity at Ulu Pandan South Works and *(B)* volatile fatty acids concentration in the primary effluent throughout the two month sampling period between the 29th May–14th August 2013. Dashed line indicates the sampling point when dissolved oxygen was decreased in the aerobic zone of Ulu Pandan South Works.

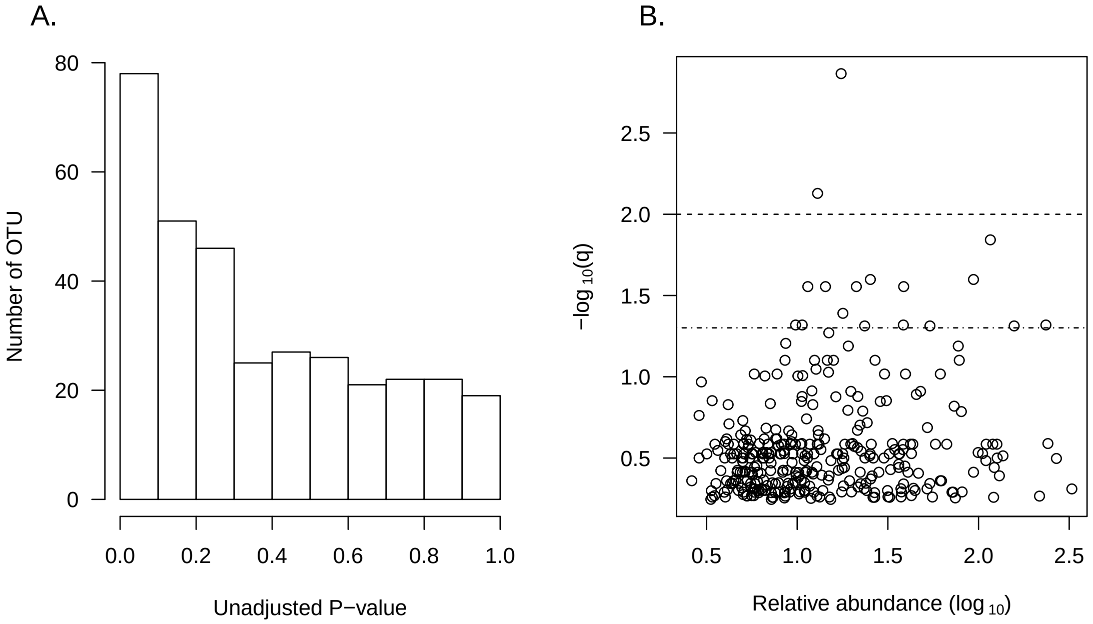

Figure S2: Additional results on signal detection for 16S data. Panel (A): histogram of raw P–values estimated from change–point model for differential abundance between anoxic and aerobic phases. Note the flat tail of this distribution is consistent with the assumptions of the Storey–Tibshirani false discovery rate estimator (see **Methods**). The proportion of taxa that show some level of statistical support for being differentially abundant in this analysis was estimated as $1 - \hat{\pi}_0 = 0.43$. Panel (B): relationship between log_{10} relative abundance (x–axis) and q–value (on $-log_{10}$ scale) for 337 OTUs detected in all 10 samples (see **Methods**. Dashed lines denote $q = 0.05$ and $q = 0.01$ respectively. Note that OTU with significant change–point statistics are distributed across the range of taxon abundance.

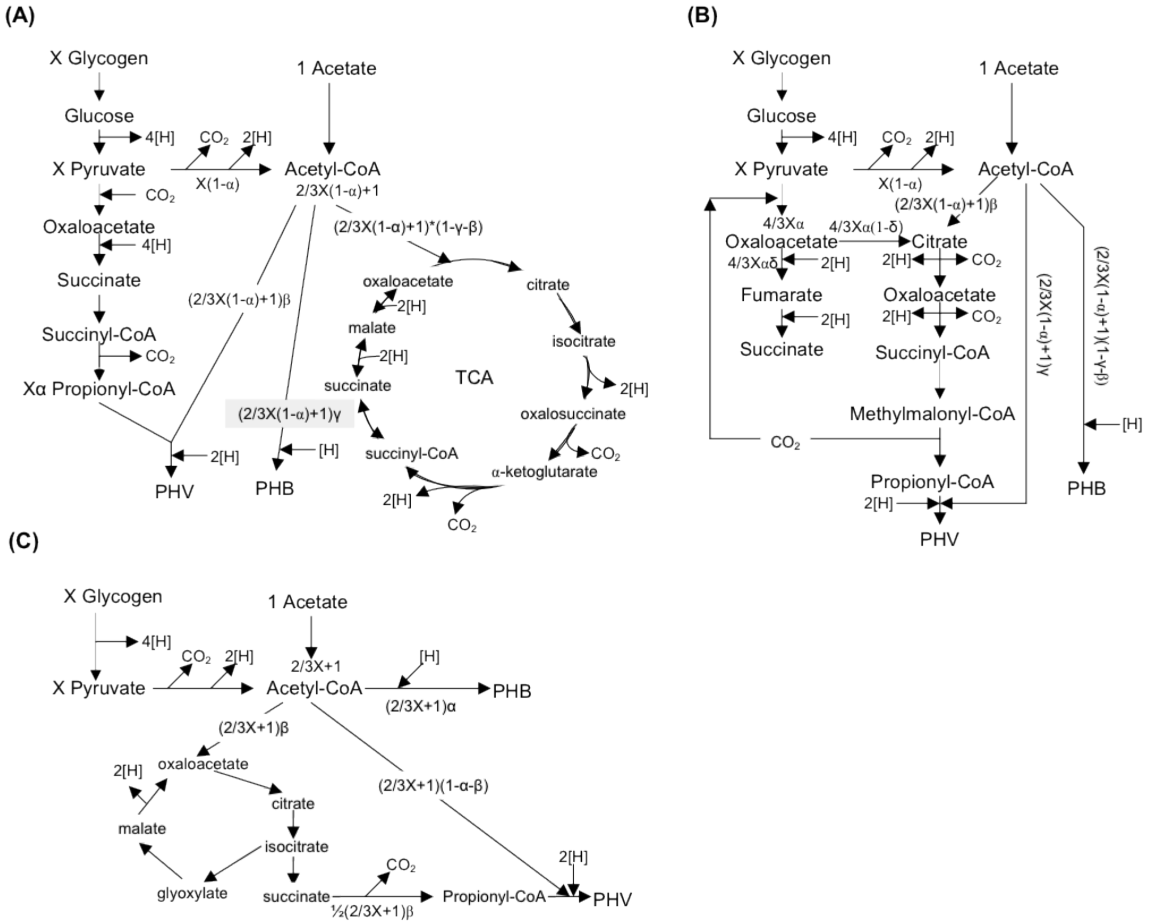

Figure S3: Anaerobic metabolism of PAO with acetate via glycolysis and TCA cycle described by *(A)*: Pereira *et al.* 1996, *(B)*: Hesselmann *et al.* 2000 and *(C)*: Yagci *et al.* 2003, figure modified from Zhou *et al.* 2009. For metabolic modelling analyses, the X parameter is the proportion of glycogen degraded per acetate consumed measured in lab–scale batch experiments and the unknown parameters α, β, γ and δ were solved as described in Zhou *et al.* 2009. The optimized model parameters are presented in **Table S3**.

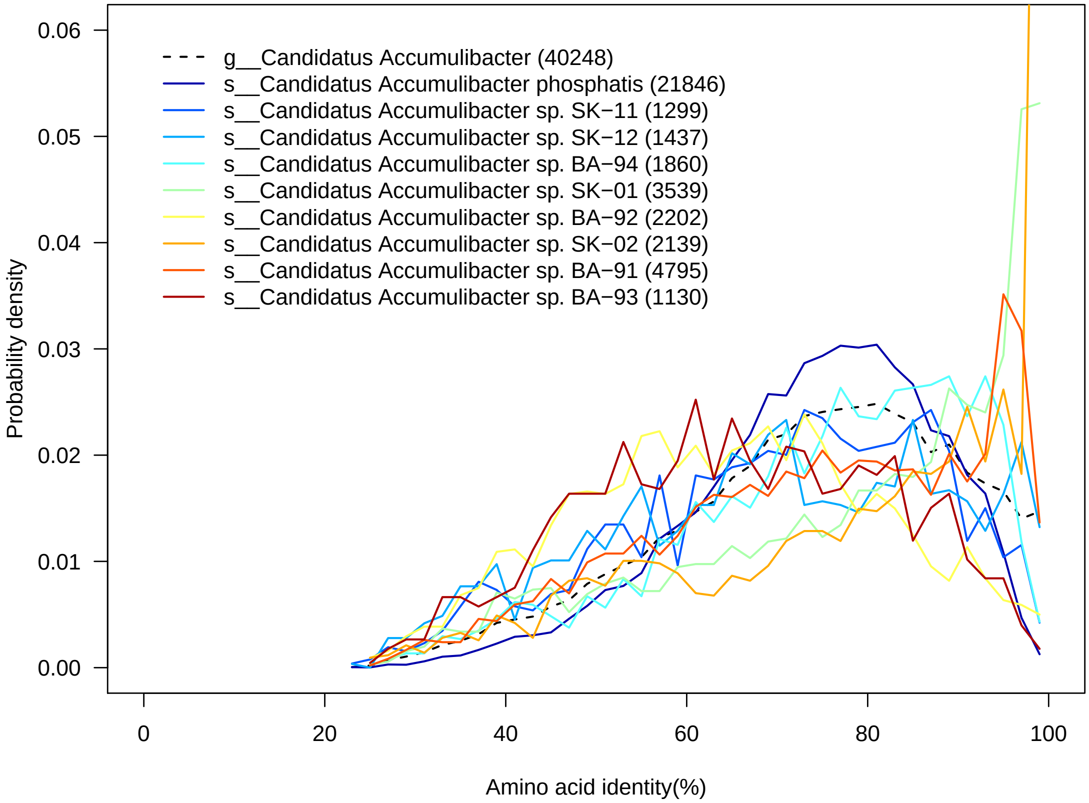

Figure S4: Distributions of amino acid identity calculated against extant Accumulibacter reference and draft genomes. See notes to **Supplementary Table 4** for details. Number in brackets denotes the number of ORFs annotated to corresponding genome at supra-threshold level in the MEGAN–LCA algorithm; note that an ORF can be annotated to multiple genomes (see **Methods**).

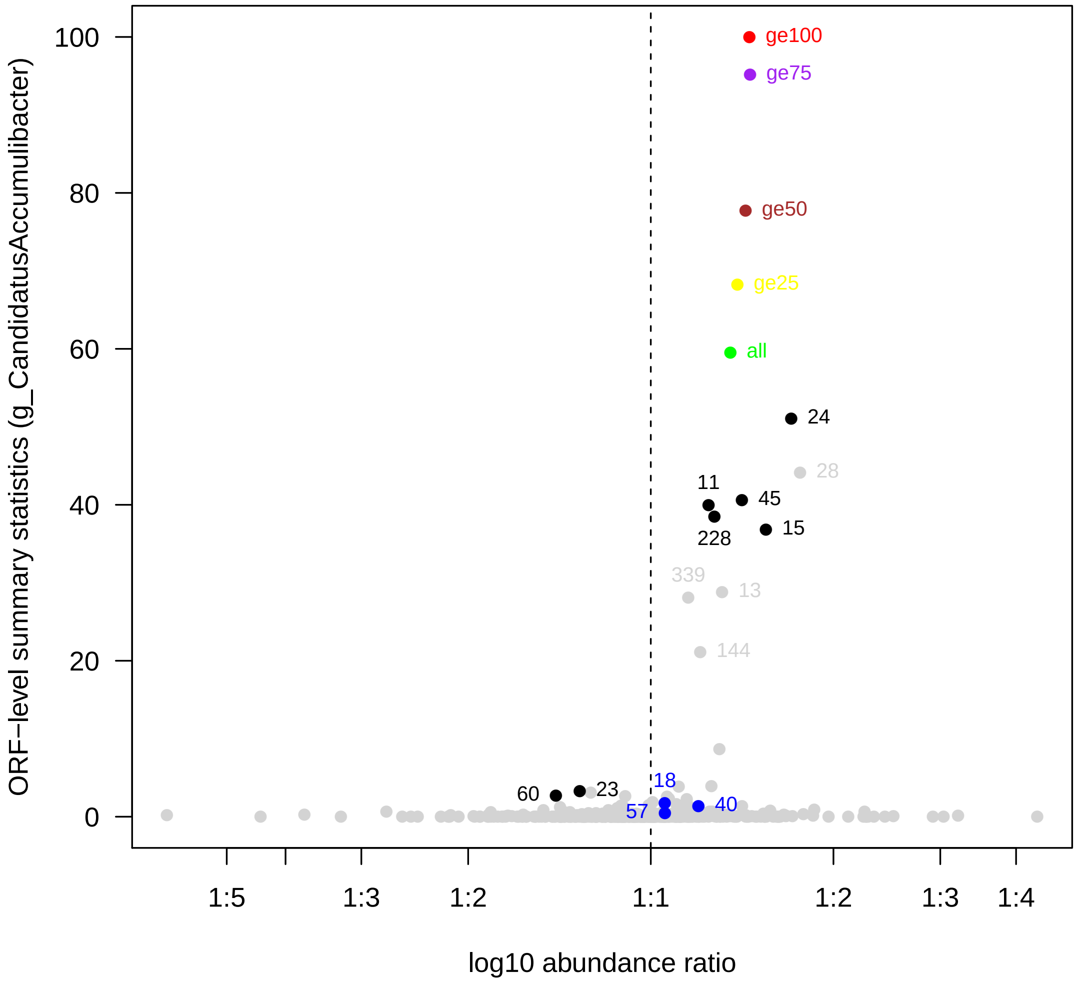

Figure S5: Differential abundance of contig–sets and MetaBAT bins between high– and low–oxygenation epochs (x–axis), and their relationship to ORF–level annotations to genus Accumulibacter (y–axis). Data points annotated to the number `num` refers to the MetaBAT–derived bin labelled `binsp1500.num` in **Supplementary Data File 1**. Data points labelled by `ge.nn` refer to sets of contigs defined by the percentage (`nn`) of their cognate ORFs that are annotated to genus Accumulibacter under . The quantity on the y–axis is calculated from Equation 5 in this document conditional on annotation to genus Accumulibacter, and the number on the x–axis is calculated from the average of Δ across all contigs included in a given contig–set or MetaBAT–derived bin.

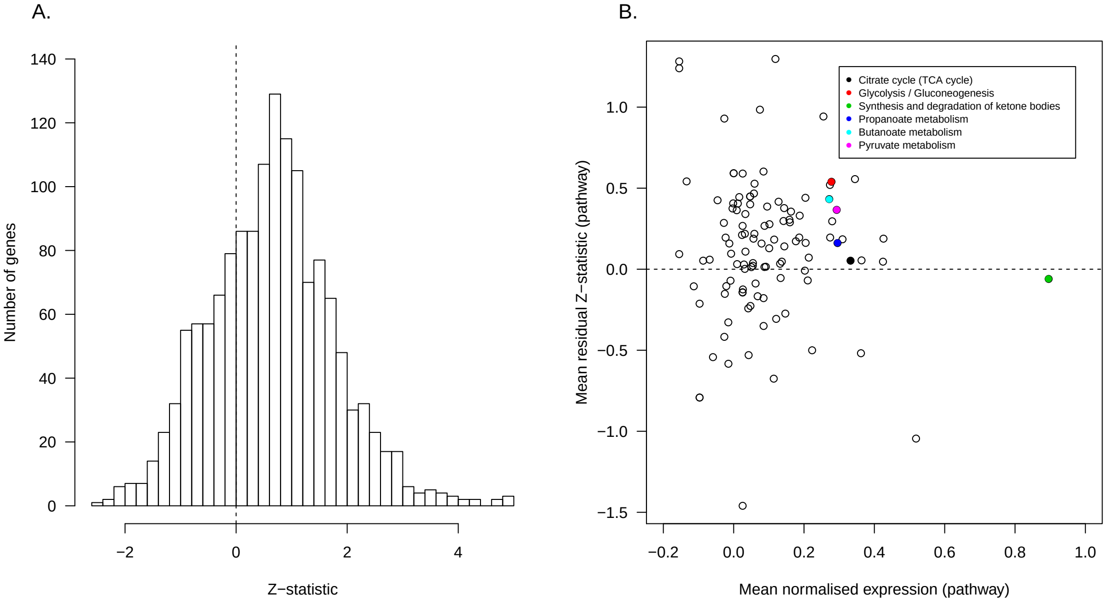

Figure S6: Panel (A): analysis of expression in genes annotated to Accumulibacter, measuring their degree of differential expression between high– and low–aeration epochs compared to all other detected genes. See **Methods** and **Supplementary Methods** for full details). Histogram of per–gene standardised statistic (Z_g) of differential expression level between high– and low–aeration epochs. Note the Z_g shows a positive bias consistent with higher level of expression in this taxa associated with increase in abundance between the high– and low–aeration epochs. Panel (B): mean per–gene standardised statistic (Z_g) of differential expression level between high– and low–aeration epochs, calculated across genes in each KEGG pathway (y–axis), plotted against the mean expression level of the pathway (x–axis). Selected pathways are highlighted.

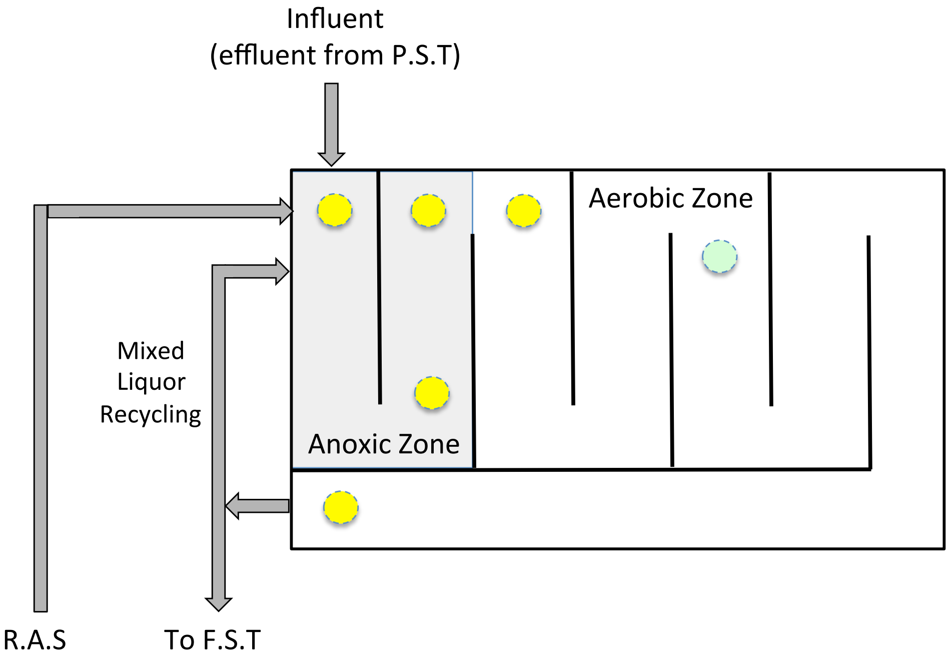

Figure S7: Schematic representation of a typical activated sludge treatment train at Ulu Pandan Water Reclamation Plant (South Works). Circles indicate sampling locations in the anoxic and aerobic zones; the light green circle, located in the mid–aerobic zone was site of sampling for gDNA and RNA samples. Abbreviations: *P.S.T*: primary sedimentation tank; *R.A.S.*: return activated sludge; *F.S.T*: final sedimentation tank.

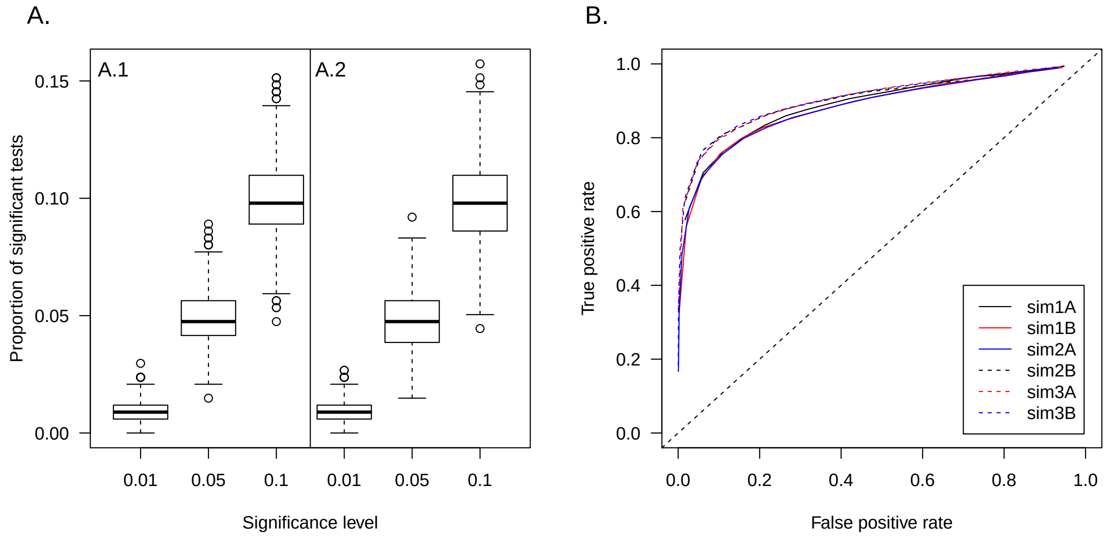

Figure S8: Results of simulation to assess signal detection properties of change–point F–statistics under conditions of the present study. *(A)*: assessment of control of false positive rate under simulated data in which all OTUs comply with the null hypothesis of no differential abundance. Boxplots show number of tests called as significant from 1000 randomised versions (resampled with replacement) of Panel (A1): the original data matrix; and A2: the SVD–filtered version of the original data that removes major time–dependent structure. (B): results of signal detection simulations plotted as true positive rate (y–axis) against false positive rate (x–axis). Data labelled A, B and C are simulations in which 20, 50 and 100 OTUs, respectively, were given non–zero abundances randomly generated from $N(0.37, 0.21)$; a distribution which approximately matched the distribution of differential abundance in observed OTUs with $q < 0.1$. Data labelled `sim1` and `sim2` are derived from the datasets used in Panel A1 and Panel A2, respectively.

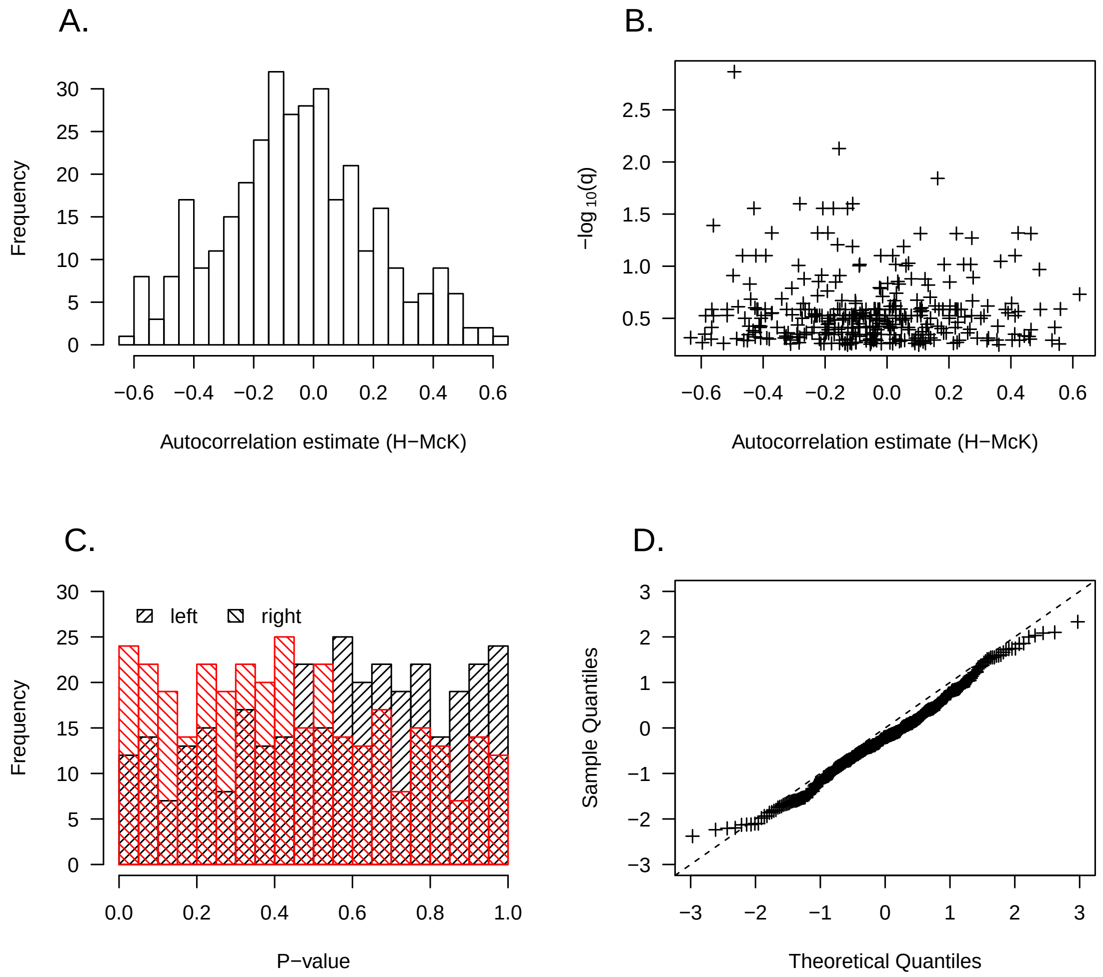

Figure S9: Analysis of possible influence of autocorrelation on test statistics. Panel (A): histogram of autocorrelation statistics for the 337 16S OTU abundance profiles calculated using the Huitema–McKean estimator; Panel (B): relationship between autocorrelation estimates and q–values (on a $-log_{10}$ scale); overall Pearson correlation is -0.03 ($P = 0.62$); Panel (C): histograms of P-values from standardised r_1+ statistic for right– and left–handed tail analyses; Panel (D): $Q - Q$ plot against of standardised r_1+ statistic against a normal distribution.
